# Gas versus GaslEss surgery for full thickness Macular hole (GEM): study protocol for a randomised, assessor-masked, surgical feasibility study

**DOI:** 10.1136/bmjophth-2025-002260

**Published:** 2025-12-25

**Authors:** George S P Murphy, Hatem A Wafa, Maryrose Tarpey, David Wyatt, Yanzhong Wang, David H Steel, Timothy L Jackson

**Affiliations:** 1 King’s College Hospital NHS Foundation Trust, London, UK; 2 School of Life Course & Population Sciences, Faculty of Life Sciences & Medicine, King’s College London, London, UK; 3 Population Health Sciences, Faculty of Life Sciences & Medicine, King's College London, London, UK; 4 Independent Research Consultant, London, UK; 5 Sunderland Eye Infirmary, Sunderland, UK; 6 Newcastle University Biosciences Institute, Newcastle upon Tyne, UK

**Keywords:** Treatment Surgery, Macula, Prospective Studies, Imaging

## Abstract

**Introduction:**

Pars plana vitrectomy with intravitreal gas tamponade has been the standard treatment for full-thickness macular holes (FTMHs) for the last 30 years. Gas tamponade has multiple drawbacks: it results in very poor vision for several weeks, limiting patients’ ability to drive and work; many undertake intensive face-down posturing for about a week after surgery, to float the gas bubble onto the macula; gas is cataractogenic and prevents adequate fundus examination; it can elevate intraocular pressure, mandating regular hospital review; patients cannot fly and the fluorocarbon tamponades are extremely potent and long-lived greenhouse gases.

Six uncontrolled retrospective case series have reported promising results utilising novel FTMH surgery without gas tamponade.

The study aims to establish if it is feasible to recruit, retain and evaluate patients with FTMHs into a pivotal randomised control trial of vitrectomy without gas tamponade. The main clinical aim is to collect preliminary safety and efficacy data, comparing gasless with standard vitrectomy.

**Methods and analysis:**

This randomised, assessor-masked, surgical, feasibility study aims to recruit 60 participants with FTMH from six English National Health Service sites. Participants will be randomised 1:1 to either standard vitrectomy, gas tamponade and postoperative posturing, or to gasless vitrectomy without posturing. Gasless surgery involves folding a hinged flap of internal limiting membrane over the hole, secured by gravity and a viscoelastic gel. Participants attend postoperatively for examination and imaging. The main feasibility outcomes are recruitment and retention rates, cross-over and participant treatment acceptability. The main clinical outcomes are primary FTMH closure (3 months), best-corrected visual acuity (6 months) and adverse events.

**Ethics and dissemination:**

South Central–Hampshire A research ethics committee gave a favourable opinion of the study (Reference: 24/SC/0019, 16 February 2024). The study results will be published in a peer-reviewed journal.

**Trial registration number:**

NCT06079593 .

**WHAT IS ALREADY KNOWN ON THIS TOPIC**

- Full-thickness macular holes can cause severe visual impairment and are currently treated with vitrectomy and gas tamponade. Gas tamponade has risks, and the associated head posturing imposes a burdensome and slow patient recovery.

**WHAT THIS STUDY ADDS**

- This is the first randomised controlled trial of macular hole surgery without gas tamponade. It will assess if a pivotal trial is feasible, inform its design and provide early efficacy and safety data.

**HOW THIS STUDY MIGHT AFFECT RESEARCH, PRACTICE OR POLICY**

- Gasless surgery would represent the largest advance in macular hole surgery for 30 years, eliminating the many downsides of gas tamponade.

## Background

Full-thickness macular holes (FTMHs) are anatomical foveal defects of all neuroretinal layers. The resulting central scotoma can cause significant visual impairment.^1^ The annual incidence is approximately 8 per 100 000 per year, with the majority occurring after age 65.^2^,^6^

Introduced in 1991, pars plana vitrectomy (PPV) with gas tamponade is now the standard treatment for FTMHs. PPV is combined with removal of the internal limiting membrane (ILM) in 95% of cases, and virtually all cases have intraocular gas tamponade.^7 8^

Recent meta-analyses reported posturing may not be necessary for FTMHs under 400 µm in diameter, but posturing is associated with improved hole closure in holes larger than 400 µm.^9^,^13^ Even if the benefit of posturing for small holes is questioned, 78% of surgeons advise it nonetheless, as the evidence is hard to interpret.^14^

There are material patient risks and burdens associated with intraocular gas. Gas absorbs spontaneously over about 4–8 weeks, but while present, it obscures vision in the affected eye and impairs binocularity, limiting patients’ ability to judge distances, drive and work.^15^ Macular hole surgery is more urgent than many elective vitreoretinal operations, as the duration of macular hole is one of the key drivers of the visual outcome. The chance of normal (20/25 or better) vision is much higher with very recent onset holes (70.6% vs 29.4%), so these cases should be treated urgently.^16^ Gas is thought to aggravate the development of post-vitrectomy cataract.^17^ Raised intraocular pressure (IOP) occurs in 15.3%–21.1% of cases.^18 19^ Gas dilution errors are rare, but can cause precipitously high IOP. Raised IOP can be painful and cause glaucomatous optic neuropathy and retinal vein occlusion.^20^ Many surgeons bring patients back early, largely to monitor IOP. Patients cannot fly, or have inhaled nitrous oxide anaesthetic until the gas absorbs.^21^ Gas can displace the intraocular lens (IOL) that is inserted during combined cataract-macular hole surgery, leading to pupil abnormalities and altered light sensitivity. Gas also makes it hard to examine the fundus, so hole closure cannot be verified (delaying repeat surgery), and other problems may go undetected.

Postoperative face-down posturing is particularly burdensome for patients.^10^ A typical regimen is face down overnight for the first night, then face down by day for 50 min/hour for 5–7 days. Thus, many patients need a carer postoperatively, many develop musculoskeletal symptoms, and some get deep vein thromboses due to immobility.^22^ Unsurprisingly, up to 50% of patients are poorly compliant with posturing, with 58% reporting that face down posturing with gas was either uncomfortable or very uncomfortable.^23^ All of our study patient involvement group described gas and postoperative posturing as the most difficult aspects of recovery following surgery.

Six recent, proof-of-concept case series reported successful FTMH surgery without the need for either posturing or intraocular gas. In two, an inverted ILM flap covered or filled the hole; the flap was kept in position using viscoelastic or autologous blood as a form of ‘glue’.^24 25^ Two series have subsequently used the viscoelastic technique,^26 27^ and another has used fibrin glue to hold the flap in situ.^28^ The final series completely removed the ILM surrounding the FTMH and used a plug of autologous blood to seal the hole.^29^ Primary closure rate was 80% of 10 patients in the fibrin glue series and ranged from 91.7% to 100% across 98 patients in the other five series, comparing favourably to current surgical success rates.^24^,^29^ The series’ report successful surgery on all sizes of FTMH, and safety was acceptable.

### Objectives

The study aims to establish if it is feasible to recruit, retain and evaluate patients with FTMHs into a pivotal randomised control trial of vitrectomy without gas tamponade. The main clinical aim is to collect preliminary safety and efficacy data, comparing gasless vitrectomy with standard vitrectomy with gas. The study also hopes to inform future trial design.

## Methods

### Trial design and setting

This is a randomised, two group, 1:1 allocated, active-control, multicentre, assessor-masked, surgical feasibility trial of approximately 36 months. Participants will be recruited and treated from approximately six National Health Service (NHS) teaching hospitals in England. The trial assesses a new surgical technique. Surgery uses an intraoperative viscoelastic, licensed as an ophthalmic viscosurgical device (OVD) and used within the scope of its indication for use. Therefore, from a UK regulatory perspective, this is not a Clinical Trial of an Investigational Medical Product.

As sites are selected, details will be available on the study record on ClinicalTrials.gov.

### Eligibility criteria

Eligibility criteria will be assessed by study ophthalmologists at participating sites based on the following criteria:

#### Inclusion criteria

- Requiring PPV to treat idiopathic (primary) FTMH.
- 18 years or older.
- Early Treatment Diabetic Retinopathy Study (ETDRS) best-corrected visual acuity (BCVA) letter score of 1 or better in the study eye.
- Able to provide written informed consent.

#### Exclusion criteria

General:

- Hypersensitivity to hyaluronate or other components of Healon Pro viscoelastic.
- Any major illness or major surgical procedure within 4 weeks.
- Any other condition that, in the opinion of the investigator, would prevent the participant from granting informed consent or complying with the protocol.

Study eye

- Previous vitreoretinal surgery, retinopexy, open-globe injury or endophthalmitis.
- Presence of fibrotic retinal proliferation or central epiretinal membrane (within 1 disc diameter of the fovea).
- Aphakia.
- Current or former myopia greater than 6 dioptres.
- Current or previous posterior uveitis or choroiditis.
- Presence of other ocular comorbidity that, in the opinion of the investigator, is likely to impair BCVA postoperatively or affect FTMH closure.
- Current ocular or periocular infection, other than mild or moderate blepharitis.
- Lens or media opacity that precludes adequate retinal assessment and imaging.

### Who will take informed consent?

Potentially eligible patients will normally be referred from their optician or other ophthalmologist to hospital vitreoretinal units, for confirmation and treatment of a FTMH. Patients will usually be approached and consented in these vitreoretinal units. A member of the research team will explain the nature of the study to participants and answer all questions regarding the study, with the aid of a patient information sheet (PIS), and give them sufficient time to decide regarding participation. Typically, this is expected to take a few days. Participants will be informed that their participation is voluntary and will be asked to sign an informed consent form (ICF) prior to any study activities. A copy of the signed ICF will be provided to all participants. An example of the PIS and ICF is included in the appendix.

### Intervention

Eligible patients will be randomised at baseline to one of two treatment arms in a 1:1 ratio through the King’s Clinical Trials Unit (KCTU)’s randomisation platform to either:

- PPV, ILM flap and viscoelastic coating without gas tamponade.
- PPV, ILM peel and 16% C2F6 gas tamponade with 5 days face down posturing.

FTMH surgery will be performed by a trial approved consultant vitreoretinal surgeon, most usually the site’s principal investigator (PI), under local anaesthesia, with or without sedation, or general anaesthesia. Surgery comprises core 23G, 25G or 27G PPV, induction of posterior vitreous detachment if required, and peripheral vitrectomy.

The surgeon’s preferred licensed macular vital stain will be used to stain the ILM.

The gasless surgical technique is based on Stopa *et al*’s description, wherein a hinged ILM flap is created under heavy liquid, then folded over the FTMH. The use of heavy liquid for flap creation is optional and based on surgeon experience and preference. A surgical video is provided in Stopa’s report.^24^

The only deviation from Stopa’s technique is that, where possible, a superior flap should be created, so that gravity helps hold it over the macula. Variations may be necessary as sometimes ILM peeling can be difficult to guide precisely (eg, the discarded flap may be used as the free cover if the folded flap does not position as desired).

A layer of Healon Pro viscoelastic will be applied to the macular area to help hold the flap in place, under heavy liquid. If necessary to retain the flap in position, the flap may be tucked into the hole. Heavy liquid will then be removed. If Healon Pro is not readily available at a study site, then an equivalent Conformité Européenne (CE)-marked OVD licensed for use in the eye may be used, subject to approval from the sponsor.

The aim is to seal the hole with ILM/viscoelastic, as our mechanistic hypothesis is that fluid flow through the hole maintains patency. However, tamponade will be used if it is not technically possible to occlude the hole using a combination of ILM and OVD.

Those randomised to standard vitrectomy will have the same surgery including a single, approximately 1 disc diameter circular ILM peel centred on the hole, but this will be discarded, and 16% C2F6 tamponade injected into the eye. ILM peeling has been chosen as the single technique for the standard of care arm, as it is still the most common technique in the UK for managing FTMHs.^30^ C_2_F_6_ has been selected as UK practice surveys indicate it is the most commonly used FTMH tamponade.^14^

In both arms, sclerotomies can be sutured if the surgeon deems it necessary.

Surgery will be video recorded (included in consent), and the duration will be recorded for all operations. Both arms will be seen 1 day, 1 week and 1, 3 and 6 months postoperatively.

Repeat surgery with vitrectomy and gas for non-closed holes will be allowed and considered a ‘failure’.

Other surgical steps will be those routinely used by the operating surgeon, or as needed to deal with any complications, such as cryotherapy and gas for a retinal tear.

#### Postoperative eye therapy

Broad-spectrum antibiotics eye drops will be prescribed for at least 1 week after surgery, and topical steroid eye drops for at least 4 weeks postoperatively. Mydriatics are allowed at the surgeon’s discretion, for approximately 1–2 weeks. The choice of steroid, antibiotic and mydriatic is at the surgeon’s preference, also considering any local policy and the particulars of each participant. Postoperative antiglaucoma medications are also permitted as required, at the surgeon’s discretion.

Patients should be provided with standard postoperative instructions and those relevant to their case, such as avoiding flying if they have gas in their eye.

#### Postoperative positioning

Participants in the standard vitrectomy arm with gas tamponade will require postoperative posturing. Participants in this arm should be advised to posture face down overnight following surgery, then face down by day for 50 mins/hour, sleeping opposite cheek to pillow, for 5 days. Participants are also advised to avoid lying on their backs or leaning backwards for 5 days after surgery. To standardise the protocol for all sites, face down posturing is being used for all hole sizes receiving gas tamponade.

Participants in the novel surgery arm will not be required to posture postoperatively.

### Relevant concomitant care permitted or prohibited during the trial

#### Cataract surgery including combined phacovitrectomy

Phacoemulsification and IOL implant can be performed prior to vitrectomy in a ‘combined procedure’ if deemed appropriate, but the decision to undertake phacoemulsification must be made and documented prior to randomisation. If an unplanned cataract occurs during surgery (eg, if there was lens touch and loss of fundal view), then this should be recorded as a deviation. Subsequent cataract surgery is allowed, but deferred until after month 3 unless clinically urgent, in which case it should be recorded as a deviation.

### Strategies to improve adherence to interventions

Participants will be provided with a standardised document detailing the postoperative posturing required for those in the arm receiving standard surgery with gas tamponade. As part of the surgical consent process, investigators are required to explain the post-operative positioning regimen.

### Outcomes

The main aim of the trial is to establish the feasibility of a subsequent pivotal trial, while also collecting provisional safety and efficacy data. To reflect this, the outcomes are divided as follows:

#### Feasibility outcomes

- Screen failure (proportion of those screened).
- Recruitment rate (participants per site per month).
- Participant retention (proportion reaching month 6 milestone).
- Cross-over (proportion converting to standard vitrectomy with gas, due to inability to seal the FTMH, or detection of peripheral retinal breaks).

#### Efficacy outcomes

- Refracted ETDRS BCVA at 6 months (mean change in ETDRS letter score from baseline).
- Surgical success (proportion with FTMH closure within 3 months without further FTMH surgery).
- Area under the BCVA versus time curve (ETDRS letter score).

#### Safety outcomes

- Adverse events (AEs).
- Intraoperative and postoperative complications.
- Development or progression of lens opacity within 6 months of vitrectomy (proportion undergoing or listed for cataract surgery and mean change in Lens Opacities Classification System 2 (LOCS2) grading).

#### Patient-reported outcomes measures

- Participant acceptability of the intervention assessed by the Macular Disease Treatment Satisfaction Questionnaire (MacTSQ; composite score).
- National Eye Institute 25-Item Visual Function Questionnaire (NEI VFQ25; composite score).
- Qualitative analysis of participant feedback.

### Participant timeline

**Figure 1:**
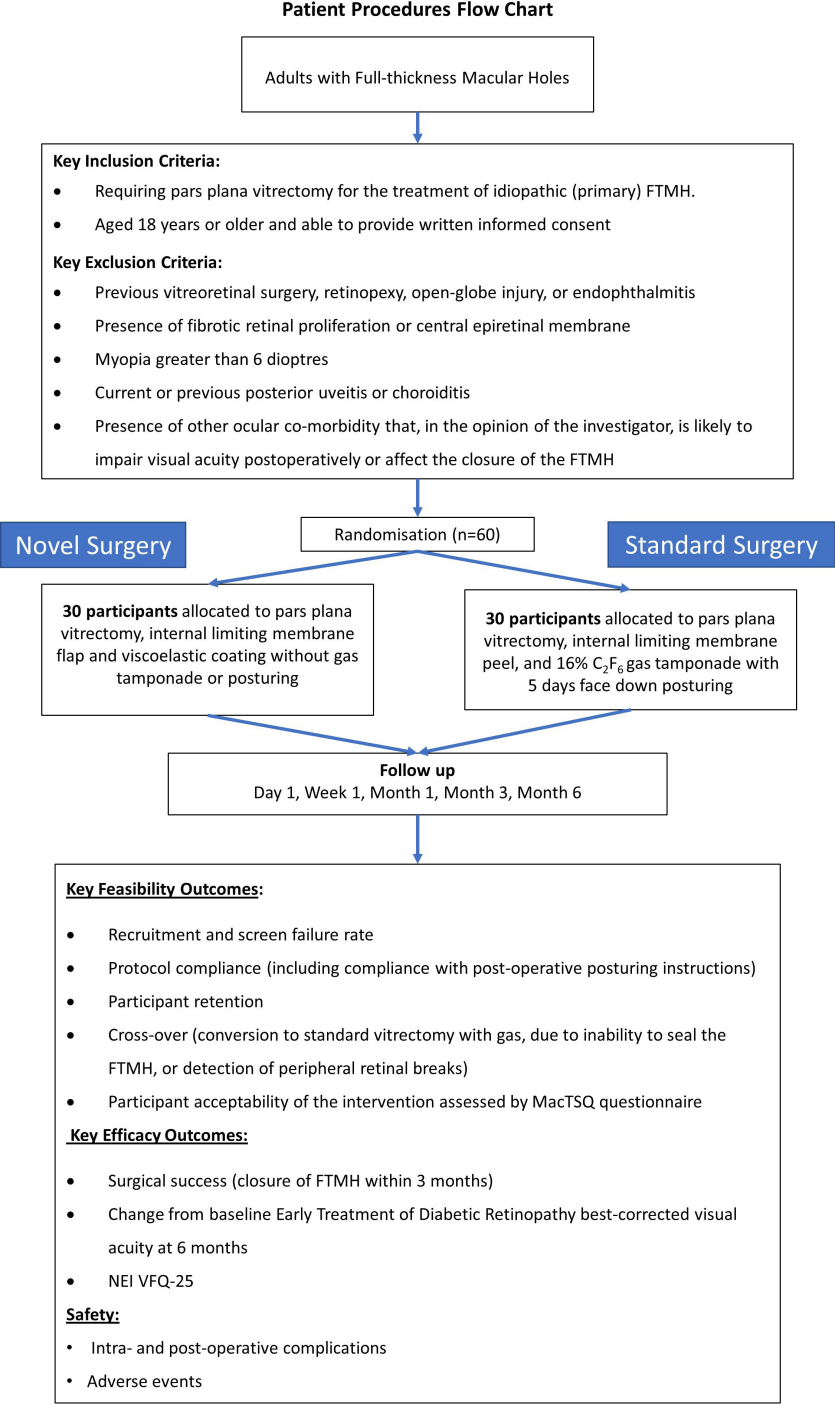
Study flow chart. C_2_F_6_, hexafluoroethane; FTMH, full-thickness macular hole; MacTSQ, Macular Disease Treatment Satisfaction Questionnaire; n, number; NEI VFQ-25, National Eye Institute 25-Item Visual Function Questionnaire.

A summary of the trial is shown in figure 1.

Screening will occur once the participant has given their written informed consent, and surgery defines the baseline visit. Participants will be randomised at the time of screening once the investigator has confirmed eligibility and a decision has been made regarding combined surgery.

Participants will be seen for follow-up according to a typical standard of care, with appointments at day 1, week 1, month 1, month 3 and month 6. A summary of the trial procedures at each appointment is included in table 1.

**Table 1:** Schedule of study assessments, summary of visit activities

Activity	Screening	Baseline	D1	W1	M1	M3	M6
Visit window (±days)	Day −42 to 0	–	0	±3	±7	±7	±7
Consent	X						
Medical and ophthalmic history	X						
Randomisation	X						
Vitrectomy		X					
Full refracted ETDRS BCVA	X					X	X
Clinic ETDRS VA*			X	X	X		
Service user questionnaire (posturing compliance)			X	X			
Macular Disease Treatment Satisfaction Questionnaire			X	X	X	X	X
Visual Function Questionnaire 25-Item	X						X
Slit-lamp examination and IOP	X		X	X	X	X	X
Lens grading	X		X	X	X	X	X
OCT (if possible)†	X		X†	X†	X	X	X
Adverse events (safety)	X	X	X	X	X	X	X
Concomitant medications	X	X	X	X	X	X	X

* Clinic ETDRS VA should be undertaken in the study eye only using an ETDRS chart with correction of any refractive error, with and without. pinhole. This should be undertaken by unmasked assessors at day 1, week 1 and month 1 in both arms. Thereafter from month 3, only suitably trained and delegated masked assessors should undertake Full refracted ETDRS BCVA.

† OCT at the ‘day 1’ and ‘week 1’ visits may not be possible in those receiving gas tamponade and may be omitted at these two visits for participants in the control arm. OCT must be performed in the study eye for all participants at all other visits, and at the day 1 and week 1 visits for those in the novel surgery arm.

BCVA, best-corrected VA; ETDRS, Early Treatment Diabetic Retinopathy Study; IOP, intraocular pressure; OCT, optical coherence tomography; VA, visual acuity; VFQ-25, National Eye Institute 25-Item Visual Function Questionnaire.

### Sample size

For feasibility and pilot studies, sample sizes between 24 and 50 have been recommended to estimate a chosen parameter.^31 32^ Using a 1:1 treatment to control ratio, a total sample size of 60 would be sufficient to estimate the SD of the outcome of at least 28 treated participants per group, with 7% attrition. We aim to estimate the screening success (assumed as 70%; 95% CI 60% to 80%) from approximately 105 potentially eligible participants.

Of the 60 participants, we will enrol at least 16 with small FTMHs (<250 µm minimum linear diameter), 16 medium (250–400 µm) and 16 large (>400 µm). We feel it is appropriate to recruit holes of varying size. This will support generalisability and provide the evidence needed to inform a future study design.

### Recruitment

With the anticipated recruitment rate of 0.5 participants/site/month, this gives an expected final patient recruited date of 23 months after trial start, in February 2026. A follow-up will finish 6 months following the recruitment of the final participant.

### Assignment of interventions

Participants will be randomised at the time of screening once the investigator has confirmed eligibility, and a decision has been made regarding combined surgery.

Participants will undergo randomisation by the process of minimisation, prior to surgery in a ratio of 1:1. They will be stratified based on planned operation (combined procedure or FTMH surgery alone) and FTMH size (small, medium and large). Phacoemulsification and IOL implant can be performed prior to vitrectomy in a ‘combined procedure’ if deemed appropriate, but the decision to undertake phacoemulsification should be made prior to randomisation.

A web-based bespoke randomisation system has been created in collaboration with the trial statisticians and the chief investigator (CI) and maintained by the KCTU for the duration of the project. It will be hosted on a dedicated server within King’s College London.

### Allocation concealment mechanism

Site-level investigators will have individual password protected access to the randomisation system. Through this, you will be informed of the study arm allocation from the central randomisation service once patients have enrolled onto the study and are determined to be eligible.

### Who will be blinded?

BCVA and central optical coherence tomography (OCT) assessors will be masked to study treatment allocation. Due to the presence of gas within the eye, it is not possible to mask participants, and while still in the eye, it is also not possible to mask BCVA assessors. Hence, the BCVA assessments on day 1, day 7 and month 1 will be performed by an unmasked assessor, as the gas will probably be present at these visits. The assessment of OCT images at a site level is not masked, but a masked grader will assess OCT images sent to an independent reading centre, to determine the OCT-based efficacy outcome measure (anatomic closure).

### Procedure for unblinding

Not applicable.

### Plans for assessment and collection of outcomes

The main feasibility outcomes will be collected through data available on the central electronic data capture (EDC) system. Sites will use corresponding paper case report forms (CRFs) to complete and upload the data onto the EDC from where it will be analysed by trial statisticians. Electronic versions of the paper CRFs will be made available to each site. Baseline demographic, ophthalmic and medical history will be recorded, including the duration of FTMH-related symptoms.

For the efficacy outcomes, full refracted best-corrected ETDRS visual acuity assessment will be performed by a qualified masked examiner. It will be performed at screening and at the postoperative visits at months 3 and 6, starting with testing at a distance of 4 m. Unmasked ETDRS visual acuity will be measured at all other visits.

OCTs will be performed at each study visit except for the day of surgery (it may not be possible to perform the postoperative OCT imaging in the control group until the gas tamponade absorbs). Heidelberg Spectralis spectral domain OCT will be used to capture a macular raster scan with a minimum size of 10^0^×20^0^, consisting of 50 sections with 60 µm spacing, centred on the fovea. An additional radial scan will be captured, consisting of 6 lines centred on the fovea.

The minimum linear diameter and base FTMH diameter will be measured with the calliper tool on the Heidelberg Eye Explorer platform, in the scan with the greatest hole width. Images will be read by the attending clinical investigator to measure the hole minimum linear and basal diameter, subsequently determine if the hole has closed, and describe the pattern of closure.^33^

Determination of development or progression of lens opacity will be measured by the mean change in LOCS2 grading.^34^

Quality of life measures will be assessed by the NEI VFQ-25 visual function questionnaire, measured at the baseline and month 6 time points. The macular treatment satisfaction questionnaire (MacTSQ; available from healthpsychologyresearch.com) will be used to assess the acceptability of treatment and will be measured at each postoperative visit.^35^

### Plans to promote participant retention

The design of the study has been chosen to replicate the normal care pathway for patients undergoing treatment for FTMH. This will limit the amount of additional burden participants will experience as part of the study and should benefit retention. Participants who chose to withdraw will be asked to complete an optional end of study visit that has all the assessments included in the month 6 visit.

### Data management

Sites will be provided with paper CRFs to complete during study visits. These correspond to a bespoke online EDC system that has been developed for the study to collect and store the relevant data. The EDC has been developed by the CI and analysts in collaboration with the KCTU. It is based on the MACRO system and is compliant with all relevant regulations. The EDC will be password-protected and reside on a secure server hosted by King’s College London.

#### Data quality processes

The CI team will undertake appropriate reviews of the entered data, in consultation with the project analyst, for the purpose of data cleaning and will request amendments as required. No data will be amended independently of the study site responsible for entering the data.

### Statistical methods

#### Statistical methods for primary and secondary outcomes

The proportion of patients who are successfully recruited after screening will be reported with 95% CIs computed by the exact binomial method, as will all outcomes assessing feasibility except for recruitment rate, which will be reported per site per month. In addition to summary statistics of the secondary outcomes (similarly to the primary and baseline outcomes), all harms and withdrawals will be reported with 95% CIs.

An exploratory efficacy analysis will be based on intent-to-treat and include all randomised participants. A secondary analysis will be per protocol comparing those who had gasless vs standard surgery, irrespective of allocation, to investigate the effect of cross-over. The safety population will include all participants who underwent randomisation.

The proportion of participants with primary surgical anatomical closure and mean BCVA (change from baseline, and area under the curve (AUC)) will be compared between groups at 6 months (Fisher’s exact test and Mann-Whitney, respectively). For the AUC analysis, VA will often be below 1 ETDRS letter when gas is in the eye, so ETDRS acuity will be converted to logarithm of the minimum angle of resolution (logMAR) with assignment of logMAR values to count fingers or worse^36^:

Count fingers at 2 ft: logMAR+1.85.

Hand motions at 2 ft: logMAR+2.3.

Visual acuity of ‘light perception’ or ‘no light perception’ will not be converted and will be reported separately.

Adherence to posturing and treatment acceptability will be described.

The MacTSQ has been validated for use in macular diseases requiring drug administration and consists of two subscales: information provision/convenience and treatment satisfaction.^35^ The individual scores from each subscale as well as the composite score will be analysed based on change from the first time point (paired sample t-test) and between groups (analysis of variance model).

Quantifiable findings from the MacTSQ will be analysed using descriptive statistics. Open-ended responses will be analysed for descriptive and analytic accounts using thematic analysis and a constant comparison approach. Analysis will pay particular attention to key factors that impact on patient experiences both positively and negatively and will integrate quantifiable data from the MacTSQ in the analysis to investigate patterns of experience within the participant population.

A detailed Statistical Analysis Plan has been prepared prior to the start of recruitment.

### Methods for additional analyses

Prespecified exploratory subgroup analyses will be performed according to the following variables:

- Operation performed; vitrectomy versus combined phacovitrectomy.
  The choice of whether to perform vitrectomy alone or combined with phacoemulsification will be examined, as the decision to remove the cataract at the same time as surgery may positively affect visual recovery and final acuity. Performing combined cataract surgery may impact on macular hole closure rates with the new technique.
- Macular hole size: small (≤250 µm), medium (251–400 µm), or large (>400 µm).
  Anatomic closure is known to be affected by the MLD of the FTMH. Outcomes will be analysed, subgrouped by categorical hole size to determine what effect it has with the novel technique.
- Lens status; phakic versus pseudophakic
  Visual outcomes may vary depending on the lens status at the beginning and end of the trial. Emerging or pre-existing cataract, if untreated, may limit BCVA improvement, compared with pseudophakic participants.
- Surgical learning curve; each surgeon’s first five cases versus subsequent cases.
  To investigate the role of the surgical learning curve on the outcome of the study, the success of the first five cases of novel gasless surgery that each surgeon performs will be compared against any subsequent cases. This will inform a descriptive analysis of the learning curve for the surgical technique, with an intention to perform a cumulative sum curve if an individual surgeon performs sufficient cases to inform the analysis.
- Vitreomacular status; vitreomacular traction versus no vitreomacular traction.
  Primary anatomical closure may be related to the VMT status at the time of surgery. Understanding the impact of VMT may help refine case selection for any subsequent pivotal trial.
- Duration of macular hole symptoms: Either more or less than the median duration, or more or less than 4 months.
  FTMH duration is known to influence both primary anatomical success and final BCVA following standard surgery. This subgroup analysis will help determine if this applies to the new surgical approach and help understand the generalisability of the results.

These factors will be applied as effects to the clinical efficacy outcomes.

### Data monitoring

A Trial Steering Committee (TSC) will be convened in accordance with National Institute for Health and Care Research (NIHR) Guidelines, and a TSC Terms of Reference. Similarly, a Data Monitoring and Ethics Committee (DMEC) will be convened per NIHR guidance and tasked to review safety data at intervals they determine as appropriate, consistent with NIHR guidance and a trial specific DMEC Charter.

### Harms

All AEs will be recorded in the CRF following consent until the patient completes the study.

All serious AEs (SAEs) (except those specified below as not requiring reporting to the Sponsor) must be recorded on an SAE form. The PI or designated individual will complete an SAE form, and it should be emailed to both the King’s College Hospital Research & Development office and to the CI within one working day of the investigator becoming aware of the event.

Reporting of safety, including all AEs, is a secondary outcome. AEs and SAEs will be reported using the Medical Dictionary for Regulatory Activities (MedDRA version 24.0).

### SAEs that do not require reporting

Failure of anatomical closure of the macular hole should be recorded on the CRF and not as an AE/SAE, unless it requires a return to theatre, in which instance it should be reported as an SAE.

IOP elevation to less than 25.0 mm Hg on the day 1 or week 1–2 postoperative visit does not need to be recorded as an AE or SAE, as this is common after vitrectomy, but the use of any pressure-lowering medicines should be recorded on the CRF. Cataract requiring surgery should be recorded as an AE.

Peripheral retinal breaks that occur during surgery should be recorded as an AE. Retinal breaks detected or emerging subsequently should also be recorded as an AE.

### Auditing

The CI will ensure there are adequate quality and number of monitoring activities conducted by the study team. This will include adherence to the protocol, procedures for consenting and ensuring adequate data quality.

The CI will inform the sponsor should he have concerns which have arisen from monitoring activities, and/or if there are problems with oversight/monitoring procedures

### Patient and public involvement

A focus group of four patients who have previously undergone FTMH surgery was convened during study development. They were asked to consider the trial design. They are also part of a larger eight-person study public (patient and carer) involvement group. Additionally, a public collaborator is part of the study team, working with the patient and public involvement (PPI) lead to co-ordinate PPI activities throughout the trial.

The group’s involvement has led to the inclusion of quality-of-life measures and qualitative feedback from carers, and to carers being invited to join the public involvement group. They have also edited and reviewed the plain English summary and patient-facing documents.

The public involvement group will further comment on how best to communicate and disseminate the results to the study participants and wider community and help to determine thresholds of success to be used in a pivotal trial.

### Protocol amendments

All protocol amendments will be prepared by the trial management team, reviewed by the sponsor and submitted to the REC for opinion. Once approved, they will be communicated to all sites, through a standard email, detailing the changes and any required steps.

### Confidentiality

Participant data will be pseudonymised. All pseudonymised data will be stored on a password-protected computer. All trial data will be stored in line with the Medicines for Human Use (Clinical Trials) Amended Regulations 2006 and the Data Protection Act.

Paper source data worksheets will be used alongside normal clinical visit data recorded on the local electronic medical record. There will be a corresponding, online, secure, eCRF. Sites will transfer data from the trial source data worksheets to the eCRF. Paper source worksheets will be stored in a secure, locked, office at each site. The data will reside on an online, secure, trial database; KCTU’s ‘Macro’ EDC.

The sponsor will archive trial data once data analysis is complete via a secure database, and the CI will act as data custodian.

### Access to data

Data will be stored by the KCTU and data extractions for monitoring will be performed on request. Following database lock and on request, KCTU will provide a copy of the final exported dataset to the CI in .csv format and the CI will onward distribute as appropriate.

At the time of consent, participants will be given the option of their anonymised study data being used for future research with third party academics. Applications for the usage of these data will be submitted to the CI and will be processed in accordance with local sponsor rules and the consent granted by participants.

It is not intended at present to host the data in an open access server following completion of the study.

### Ancillary and post-trial care

Following their exit from the study, participants will not normally require further ophthalmic input. It is therefore anticipated that most participants will be discharged for regular optician review. If there is a need for further management, this will be under NHS standard care. The Sponsor has indemnity to cover negligent harm to trial participants in the UK.

### Dissemination policy

Working with the study team, the public (patient and carer) contributor panel will lead on dissemination of the trial’s outputs, including participants’ reported outcomes, to inform a wider audience. This will include producing a lay summary report for patients, shared through eye charities and hospital news channels as well as information hosted on appropriate websites and social media. Participants will be informed of the trial’s progress via a regular newsletter.

All participants will be invited to receive a copy of the trial results (unless they wish not to).

Vitreoretinal surgeons are key stakeholders, as they will be the ones to recruit onto any subsequent pivotal trial and drive adoption if gasless macular hole surgery is proven efficacious. Accordingly, the study results will be presented at national and international retinal conferences and submitted for publication in a peer-reviewed clinical journal. Authorship will be determined by the CI, in discussion with the coinvestigators, to reflect the relative contribution of staff to the design, execution, analysis and write-up of the results. It is anticipated that the coinvestigators will be named authors. It is not expected that professional writers will be employed. The full protocol and Statistical Analysis Plan will be submitted for public record alongside the outcome paper.

## Discussion

There have been incremental advances in surgery for patients with FTMH, including the introduction of small gauge vitrectomy, vital staining of the ILM and more efficient vitrectors. While the anatomic and visual success of the operation has increased, there are still risks and drawbacks to relying on tamponade. Eliminating gas would likely be the most important advance in FTMH surgery since 1991, with the potential to deliver material patient-focused benefit, accelerating postoperative visual recovery and function, and removing the need to posture. It will also potentially benefit the healthcare system and will deliver important environmental benefits, as the tamponade agents are potent and enormously long-lasting greenhouse gases, with atmospheric lifetimes from 2600 to 10 000 years.^37^

Deciding which OVD to use considers what is readily available, with a good safety profile, low cost and is already licenced to be used in the posterior segment, as these factors will help to accelerate the adoption of any new technique, if proven effective. Although the OVD used in this study is licensed for use in the posterior segment of the eye, most viscolestics are not. Those used in case series and reports were used off licence, but that may not necessarily be a barrier to their adoption, as clinicians are very familiar with OVDs, and they have a very acceptable and well-characterised safety profile. However, using an unlicensed OVD within the trial would impose regulatory barriers that will make the trial harder to deliver, so we restricted use to one agent, but considered any others that emerge and have suitable licence (indication for use).

There is no one feasibility outcome threshold that would necessarily determine if a pivotal trial is appropriate. Instead, an in toto evaluation of the results may be more informative. For example, if recruitment was slow, with effectiveness only apparent below a certain hole size, then that might argue not to proceed to a pivotal trial. However, if the results also indicated that treatment appears very effective in this subgroup, with no safety concerns, and gasless surgery was greatly favoured by participants, then a long trial across many international sites may still be justified, given the potential clinical impact.

The outcomes of the study should determine if a pivotal study is both feasible and appears worthwhile, and help inform its design. If such a future trial shows gasless surgery is safe and effective, this could deliver material patient benefits with relatively few barriers to adoption and global impact.

## Supplementary material

10.1136/bmjophth-2025-002260 online supplemental file 1

## Data Availability

No data are available.
