# Supplementary material for: Gas versus GaslEss surgery for full thickness Macular hole (GEM): study protocol for a randomised, assessor-masked, surgical feasibility study
Source: BMJ Open Ophthalmol. 2025 Dec 25;10(1):e002260. doi: 10.1136/bmjophth-2025-002260 (PMC12742066; doi:10.1136/bmjophth-2025-002260)
Supplement: online supplemental file 1 [file bmjophth-10-1-s001.pdf]

GEM

GaslEss Macular hole surgery (GEM): a feasibility study

**Chief Investigator**	King's College Hospital<br>Denmark Hill<br>London SE5 9RS<br><br>Tel:<br>Email:
**Co-Investigator**	King's College Hospital<br>Denmark Hill<br>London SE5 9RS<br><br>Tel:<br>Email:
**Sponsor**	Research & Development Manager<br>The R&D Office,<br>First Floor Coldharbour Works,<br>245A Coldharbour Lane,<br>Brixton,<br>London SW9 8RR
**Funder:**	National Institute for Health and Care Research(NIHR): Research for Patient Benefit (RfPB) Programme: NIHR204994
**IRAS Reference**	321486
**Clinical Trials Reference**	NCT06079593

Protocol Version and Date V1.3 27/06/2024

**Previous protocol versions**		
**Versions No**	**Version Date**	**Status**
1.0		
1.1	27/Mar/2024	Changes:<br>Section 6.4 (recruitment): Legend corrected on graph to describe the two groups presented.<br>Section 13.2 KCTU Randomisation: Correction of method of randomisation from 'block' to 'minimisation'.<br>Section 15.1 SAE definition: Correction to remove 'vision-threatening' from definition of SAE.<br>Section 15.2.2 Causality: Option of not-assessable removed<br>Section 15.5 Definition of SAEs that do not require reporting: Correction that 'cataract surgery' should be recorded as an AE.<br>Section 15.5 Definition of SAEs that do not require reporting: Correction that intraoperative retinal breaks should be recorded as an AE.
1.2	24/Apr/2024	Section 6.1 Screening: removal of word 'simple' and clarification that randomisation will be done by minimisation.<br>Section 9: RECRUITMENT & RANDOMISATION: Correction of 'block randomisation' to 'minimisation'<br>Section 13.2: KCTU Randomisation: Correction of two instances of 'block randomisation' to 'minimisation'
1.3	27/Jun/2024	Section 1: Table 1 schedule of study assessments;<br>-Screening period increased to up to 42 days prior to baseline date.<br>-Removal of "Full refracted ETDRS BCVA" at the Day 1, week 1, and month 1 visits.<br>-Addition of "Clinic ETDRS VA" at the Day 1, week 1, and month 1 visits.<br>-Footnote clarifying that Clinic ETDRS VA is to be performed by an unmasked assessor, and Full refracted ETDRS BCVA is to be undertaken by a masked assessor.<br>-Footnote clarifying that omission of the OCT is permitted at the day 1 and week 1 visit for participants receiving gas tamponade.<br>Section 5.5: Macular hole surgery: correction of spelling of "principal"<br>Section 5.5: Macular hole surgery: Clarification that cataract surgery and<br>Section 5.6: Cataract surgery: Clarification that appropriately qualified delegated surgeons can perform cataract surgery.<br>Section 6.2: Baseline: Clarification that the macular hole surgery will be performed by a consultant vitreoretinal surgeon. Allowing the specificity in sections 5.5 and 5.6 for parts of the surgery to be performed by suitably qualified surgeons.<br>Section 6.3: Day 1, Week 1 & Month 1,3,6 Visits: Updated to reflect that ETDRS VA rather than BCVA will be performed at each study visit. Clarification that masked refracted BCVA will be undertaken at months 3, and 6. Standard clinic ETDRS visual acuity, with usual distance correction if appropriate, will be undertaken at the day 1, week 1, and month 1 visits.<br>Section 17: Training:<br>Clarification that it is the macular hole part of the surgery which requires the surgeon to be a consultant vitreoretinal surgeon.<br>Clarification that the refracted best corrected visual acuity assessors are masked.

		Added clarification that the standard clinic ETDRS VA may be undertaken by suitably trained clinic staff but must still be appropriately delegated.

CONTENTS

1 Table 1: Schedule of Study Assessments 10
2 INTRODUCTION 11
3 BACKGROUND AND RATIONALE 11
4 OBJECTIVES & OUTCOMES 13
4.1 Feasibility outcomes 13
4.2 Efficacy outcomes 13
4.3 Safety outcomes 13
4.4 Patient reported outcomes measures 14
5 STUDY OVERVIEW 14
5.1 Design and setting 14
5.2 End of study 14
5.3 Trial Flowchart 15
5.4 Study timeline 16
5.5 Macular hole surgery 17
5.5.1 Post-operative head positioning (posturing) 18
5.5.2 Post-operative management 18
5.6 Cataract surgery 19
5.7 Data Capture 19
5.8 OVD risks 19
6 STUDY SCHEDULE 20
6.1 Screening 20
6.1.1 Optical Coherence Tomography (OCT) 21
6.1.2 OCT analysis 21
6.2 Baseline 22
6.3 Day 1, Week 1 & Month 1,3,6 Visits 22
6.4 Recruitment timelines and trial duration 23
6.5 Study Participation Discontinuation 24
7 CONSENT 24
8 ELIGIBILITY CRITERIA 25
8.1 Inclusion Criteria 25
8.2 Exclusion Criteria 25
9 RECRUITMENT & RANDOMISATION 26
10 STATISTICAL METHODS 27
10.1 Sample size 27

10.2 Statistical Analysis 27
11 PATIENT AND PUBLIC INVOLVEMENT (PPI) 29
12 FUNDING AND SUPPLY OF EQUIPMENT 30
13 DATA HANDLING AND MANAGEMENT 30
13.1 Macro EDC 30
13.2 KCTU Randomisation 32
13.3 Trial Management 33
13.4 Ethics / Regulatory Approvals 33
14 PEER AND REGULATORY REVIEW 34
15 ADVERSE EVENTS AND INCIDENT REPORTING 34
15.1 Definitions of Adverse Events 35
15.2 Assessments of Adverse Events 35
15.2.1 Severity 35
15.2.2 Causality 35
15.2.3 Expectedness 36
15.3 Procedures for recording adverse events 36
15.4 Procedures for recording and reporting serious adverse events 36
15.5 Serious Adverse Events that do not require reporting 37
15.6 Reporting Urgent Safety Measures 37
15.7 Protocol deviations and notification of protocol violations 37
15.8 Trust incidents and near misses 40
16 MONITORING AND AUDITING 40
17 TRAINING 40
18 INTELLECTUAL PROPERTY 41
19 INDEMNITY ARRANGEMENTS 41
20 ARCHIVING 42
21 PUBLICATION AND DISSEMINATION POLICY 42
22 REFERENCES 43
23 APPENDICES 44

LIST OF ABBREVIATIONS

95% CI	95% Confidence Interval
AE	Adverse Event
ADE	Adverse Device Effect
AR	Adverse Reaction
AUC	Area Under the Curve
BCVA	Best Corrected Visual Acuity
C_2F_6	Hexafluoroethane
CE	Conformité Européenne
CI	Chief Investigator
CRF	Case Report Form
CTIMP	Clinical Trial of Investigational Medical Product
CTU	Clinical Trials Unit
DMEC	Data Monitoring and Ethics Committee
eCRF	Electronic Case Report Form
EDC	Electronic Data Capture
EMR	Electronic Medical Record
ETDRS	Early Treatment Diabetic Retinopathy Study
FDA	Food and Drug Administration
FTMH	Full-Thickness Macular Hole
GCP	Good Clinical Practice
HRA	Health Research Authority
ICF	Informed Consent Form
ILM	Internal Limiting Membrane
IOL	Intraocular Lens
IOP	Intraocular Pressure
KCH	King's College Hospital
KCTU	King's Clinical Trials Unit
KHP-CTO	King's Health Partners Clinical Trials Office
LOCS2	Lens Opacities Classification System 2
logMAR	Logarithm of the Minimum Angle of Resolution
MacTSQ	Macular Disease Treatment Satisfaction Questionnaire
MedDRA	Medical Dictionary for Regulatory Activities
MHRA	Medicines and Healthcare products Regulatory Agency
NEI VFQ25	National Eye Institute 25-Item Visual Function Questionnaire
NICE	National Institute for health and Clinical Excellence
NIHR	National Institute for Health and Care Research
NHS	National Health Service
OCT	Optical Coherence Tomography
OVD	Ophthalmic Viscoelastic Device
PI	Principal Investigator
PIS	Participant Information Sheet
PPI	Patient and Public Involvement
PPV	Pars Plana Vitrectomy
PRN	Pro re nata (as required)
PVD	Posterior Vitreous Detachment
RCT	Randomised Controlled Trial
R&D	Research and Development
REC	Research Ethics Committee
SAE	Serious Adverse Event
SADE	Serious Adverse Device Event

SAR	Serious Adverse Reaction
SOP	Standard Operating Procedure
SUSAR	Suspected Unexpected Serious Adverse Reaction
TMF	Trial Master File
TSC	Trial Steering Committee
UADE	Unanticipated Adverse Device Effect
UAR	Unexpected Adverse Reaction
VA	Visual Acuity

STUDY SUMMARY

STUDY OVERVIEW	
Full title	GaslEss Macular hole surgery (GEM): a feasibility study
Objectives	To establish if it is feasible to recruit, retain, and evaluate patients with full-thickness macular holes (FTMHs) into a larger randomised controlled trial of vitrectomy without gas tamponade.<br><br>The main clinical aim is to collect preliminary safety and efficacy data, comparing gasless vitrectomy with standard vitrectomy with gas.
Type of trial	Randomised, multi-centre, observer-masked, surgical, device feasibility trial
Health condition(s) or problem(s) studied	FTMH
Target sample size	60 participants
Trial duration per participant	6 months from surgery to final follow up
Main inclusion criteria	• Requiring pars plana vitrectomy to treat idiopathic (primary) FTMH.<br>• 18 years or older<br>• ETDRS best-corrected visual acuity (BCVA) letter score of 1 or better in the study eye
Main exclusion criteria	**General:**<br>• Hypersensitivity to hyaluronate or other components of Healon Pro® viscoelastic<br>• Any major illness or major surgical procedure within 4 weeks<br>• Any other condition that, in the opinion of the investigator, would prevent the participant from granting informed consent or complying with the protocol<br>**Study eye:**<br>• Previous vitreoretinal surgery, retinopexy, open-globe injury, or endophthalmitis<br>• Presence of fibrotic retinal proliferation or central epiretinal membrane within 1 disc diameter of the fovea<br>• Aphakia<br>• Current or former myopia greater than 6 dioptres<br>• Current or previous posterior uveitis or choroiditis<br>• Presence of other ocular co-morbidity that, in the opinion of the investigator, is likely to impair BCVA postoperatively or affect FTMH closure<br>• Current ocular or periocular infection, other than mild or moderate blepharitis<br>• Lens or media opacity that precludes adequate retinal assessment and imaging
Statistical methodology and analysis	Data analysis will be largely descriptive, summarising the feasibility outcomes.<br>An exploratory efficacy analysis will be intent-to-treat and include all randomised participants. In case of cross-over, a secondary analysis will compare those who had gasless versus standard surgery,

	regardless of randomisation. The safety population will include all randomised participants.
**STUDY TIMELINES**	
Study duration/length	36 months
Expected start date	1st November 2023
End of study definition and anticipated date	Set at 6 months following the final visit of the final participant, anticipated to be June 2026
Key study milestones	Sep 2023<br>Apply to research ethics committee (REC)<br><br>Feb 2023 – Oct 2023<br>Local research and development approvals<br><br>Sep 2023 – Feb 2024<br>Contracting with subsites & study sites set-up<br><br>Nov 2023 – Oct 2025<br>Recruitment<br><br>Oct 2025 – Apr 2026<br>Follow up<br><br>Apr 2026 – Sep 2026<br>Data cleaning and database lock<br><br>Feb 2026 – Oct 2026<br>Data analysis<br>Manuscript preparation
Research ethics committee number	24/SC/0019
Protocol Version Number	1.3

1 Table 1: Schedule of Study Assessments

Summary of visit activities

Activity	Screening	Baseline	D1	W1	M1	M3	M6
Visit window (±days)	Day -42 to 0	-	0	±3	±7	±7	±7
Consent	X						
Medical and ophthalmic history	X						
Randomisation	X						
Vitrectomy		X					
Full refracted ETDRS BCVA	X					X	X
Clinic ETDRS VA±			X	X	X		
Service user questionnaire (posturing compliance)			X	X			
Macular Disease Treatment Satisfaction Questionnaire (MacTSQ)			X	X	X	X	X
Visual function questionnaire (VFQ-25)	X						X
Slit-lamp examination and IOP	X		X	X	X	X	X
Lens grading	X		X	X	X	X	X
OCT (if possible*)	X		X*	X*	X	X	X
Adverse events (safety)	X	X	X	X	X	X	X
Concomitant medications	X	X	X	X	X	X	X

±Clinic ETDRS VA should be undertaken in the study eye only using an ETDRS chart with correction of any refractive error, with and without pinhole. This should be undertaken by unmasked assessors at D1, W1 and M1 in both arms. Thereafter from M3, only suitably trained and delegated masked assessors should undertake Full refracted ETDRS BCVA

* OCT at the ‘Day 1’ and ‘Week 1’ visits may not be possible in those receiving gas tamponade and may be omitted at these two visits for participants in the control arm. OCT must be performed in the study eye for all participants at all other visits, and at the day 1 and week 1 visits for those in the novel surgery arm.

2 INTRODUCTION

This randomised feasibility study will investigate a novel surgical technique for treating full-thickness macular holes (FTMHs), without the need for the gas tamponade that is usually used to fill the vitreous cavity of the eye. If feasibility is established, this would guide a larger definitive study.

Direct benefits to patients include improved speed of visual recovery, avoidance of head positioning after surgery, and decreased risk of raised intraocular pressure (IOP). The device being used is Healon Pro OVD® (Johnson & Johnson UK), a CE marked device that has been used for intraocular surgery for 40 years. Healon Pro® OVD will be used in accordance with its indication for use.

3 BACKGROUND AND RATIONALE

FTMHs are an anatomical foveal defect of all neuroretinal layers. The resulting central scotoma can cause significant visual impairment.[1] The annual incidence is approximately 8 per 100,000 per year, with the majority occurring after age 65.[2-6]

Introduced in 1991, pars plana vitrectomy (PPV) with gas tamponade is now the standard treatment for FTMHs. PPV is combined with removal of the internal limiting membrane (ILM) in 95% of cases, and virtually all cases have intraocular gas tamponade.[7,8]

Recent meta-analyses reported posturing may not be necessary for FTMHs under 400 microns in diameter, but posturing is associated with improved hole closure in holes larger than 400 microns.[9-11] Even if the benefit of posturing for small holes is questioned, 78% of surgeons advise it nonetheless, as the evidence is hard to interpret.[12]

There are material patient risks and burdens associated with intraocular gas. Gas absorbs spontaneously over about 4-8 weeks, but whilst present it obscures vision in the affected eye and impairs binocularity, limiting patient's ability to judge distances, drive and work.[13] Gas aggravates the development of post-vitrectomy cataract, and raised IOP occurs in up to 59% of cases.[14] Gas dilution errors are rare, but can cause precipitously high IOP. Raised IOP can be painful, and cause glaucomatous optic neuropathy and retinal vein occlusion.[14] Many surgeons bring patients back early, largely to monitor IOP. Patients cannot fly, ascend above 300 metres,

or have inhaled nitrous oxide anaesthetic until the gas absorbs.[15] Gas can displace the intraocular lens (IOL) that is inserted during combined cataract-macular hole surgery, leading to pupil abnormalities and altered light-sensitivity. Gas also makes it hard to examine the fundus, so hole closure cannot be verified (delaying repeat surgery), and other problems may go undetected.

Post-operative face-down posturing is particularly burdensome for patients.[10] A typical regimen is face down overnight for the first night, then face down by day for 50 minutes/hour for 5-7 days. Thus, many patients need a carer post-operatively, many develop musculoskeletal symptoms, and some get deep vein thromboses due to immobility.[16] Unsurprisingly, up to 50% of patients are poorly compliant with posturing.[17] Recent meta-analyses reported posturing may not be necessary for FTMHs under 400 microns in diameter, but posturing is associated with improved hole closure in holes larger than 400 microns.[9-11] Even if the benefit of posturing for small holes is questioned, 78% of surgeons advise it nonetheless, as the evidence is hard to interpret.[12]

Three recent, proof-of-concept case series reported successful FTMH surgery without the need for either posturing or intraocular gas. In two, an inverted ILM flap covered or filled the hole; the flap was kept in position using viscoelastic or autologous blood as a form of 'glue'.[18,19] The third series completely removed the ILM surrounding the FTMH and used a plug of autologous blood to seal the hole.[20] Primary closure rates ranged from 91.7% to 100% across 62 patients, comparing favourably to current surgical success rates.[18-20] Safety was favourable.

We aim to complete a feasibility study of gasless FTMH surgery, using a modified ILM-flap, viscoelastic, and no-posturing approach. The surgical technique is based on Stopa *et al*'s [2020] description, wherein following PPV a hinged ILM flap is created under heavy liquid, then folded over the FTMH. The surgeon should use decalin (Eftiar® Decalin, Perfluoro-Decalin, DORC, Zuidland, The Netherlands) if heavy liquid is utilised during surgery, in accordance with its marketing authorisation, although the use of heavy liquid is optional and based on surgeon experience and preference. A surgical video is provided in Stopa's report:

https://cdn-links.lww.com/permalink/iae/b/iae_2020_04_21_stopa_220-0241_sdc1.mp4

Following this, viscoelastic will be applied to 'glue' the ILM flap in place, and no intraocular tamponade will be used. This will be compared with the current standard of care; PPV, ILM peeling, intraocular gas tamponade with 16% C_2F_6, and 5 days of postoperative posturing.

We have selected a licensed ophthalmic hyaluronate-based viscoelastic (Healon PRO®, Johnson & Johnson Medical LTD, UK) as our tissue 'glue', as it is thought to be safe (viscoelastics are used

in most eye operations), is widely available, and does not have the patient/staff/regulatory burdens associated with blood or serum products.

The results of this study will help to determine if a further study is deliverable. If so, then we aim to inform the trial's design, including estimated effect size, sample size calculation, selection of participants, outcome measures and recruitment timelines.

4 OBJECTIVES & OUTCOMES

The main feasibility aim is to establish if it is feasible to recruit, retain, and evaluate patients with FTMHs into a pivotal RCT of vitrectomy without gas tamponade.

The main clinical aim is to estimate the efficacy of gasless surgery compared to standard PPV, and collect preliminary safety data.

4.1 Feasibility outcomes

- Screen failure (proportion of those screened)
- Recruitment rate (participants per site per month)
- Participant retention (proportion reaching month 6 milestone)
- Cross-over (proportion converting to standard vitrectomy with gas, due to inability to seal the FTMH, or detection of peripheral retinal breaks)

4.2 Efficacy outcomes

- Early Treatment Diabetic Retinopathy Study (ETDRS) best-corrected visual acuity (BCVA) at 6 months (mean change from baseline)
- Surgical success (proportion with FTMH closure within 3 months, without further FTMH surgery)
- Area under the BCVA versus time curve (ETDRS letter score)

4.3 Safety outcomes

- Adverse events (AEs)
- Intra- and post-operative complications
- Development or progression of lens opacity within 6 months of vitrectomy (proportion undergoing or listed for cataract surgery and mean change in Lens Opacities Classification System 2 [LOCS2] grading)

4.4 Patient reported outcomes measures

- Participant acceptability of the intervention assessed by the Macular Disease Treatment Satisfaction Questionnaire (MacTSQ; composite score)
- National Eye Institute 25-Item Visual Function Questionnaire (NEI VFQ25; composite score)
- Qualitative analysis (please see Statistical Analysis section)

5 STUDY OVERVIEW

5.1 Design and setting

This is a randomised, multi-centre, observer-masked, surgical device feasibility trial of approximately 36 months. Participants will be recruited and treated from approximately six teaching hospitals: King's College Hospital; Sunderland Eye Infirmary; Bristol Eye Hospital; Hull and East Yorkshire Eye Hospital; St Paul's Eye Unit Liverpool; and the Sussex Eye Hospital.

Surgery utilises an established intraoperative viscoelastic that is licensed as a device and is used within the scope of its indication for use.

Eligible patients will be randomised at baseline to one of two treatment arms in a 1:1 ratio through the King's Clinical Trials Unit (CTU)'s randomisation platform to either:

- PPV, ILM flap and viscoelastic coating without gas tamponade
- PPV, ILM peel, and 16% C_2F_6 gas tamponade with 5 days face down posturing

During study visits participants will undergo assessments as outlined in Table-1.

Section 5.3 below provides an outline of the overall study design.

5.2 End of study

The end of study is set at 6 months following the final visit of the final participant, anticipated to be about June 2026

5.3 Trial Flowchart

Patient Procedures Flow Chart

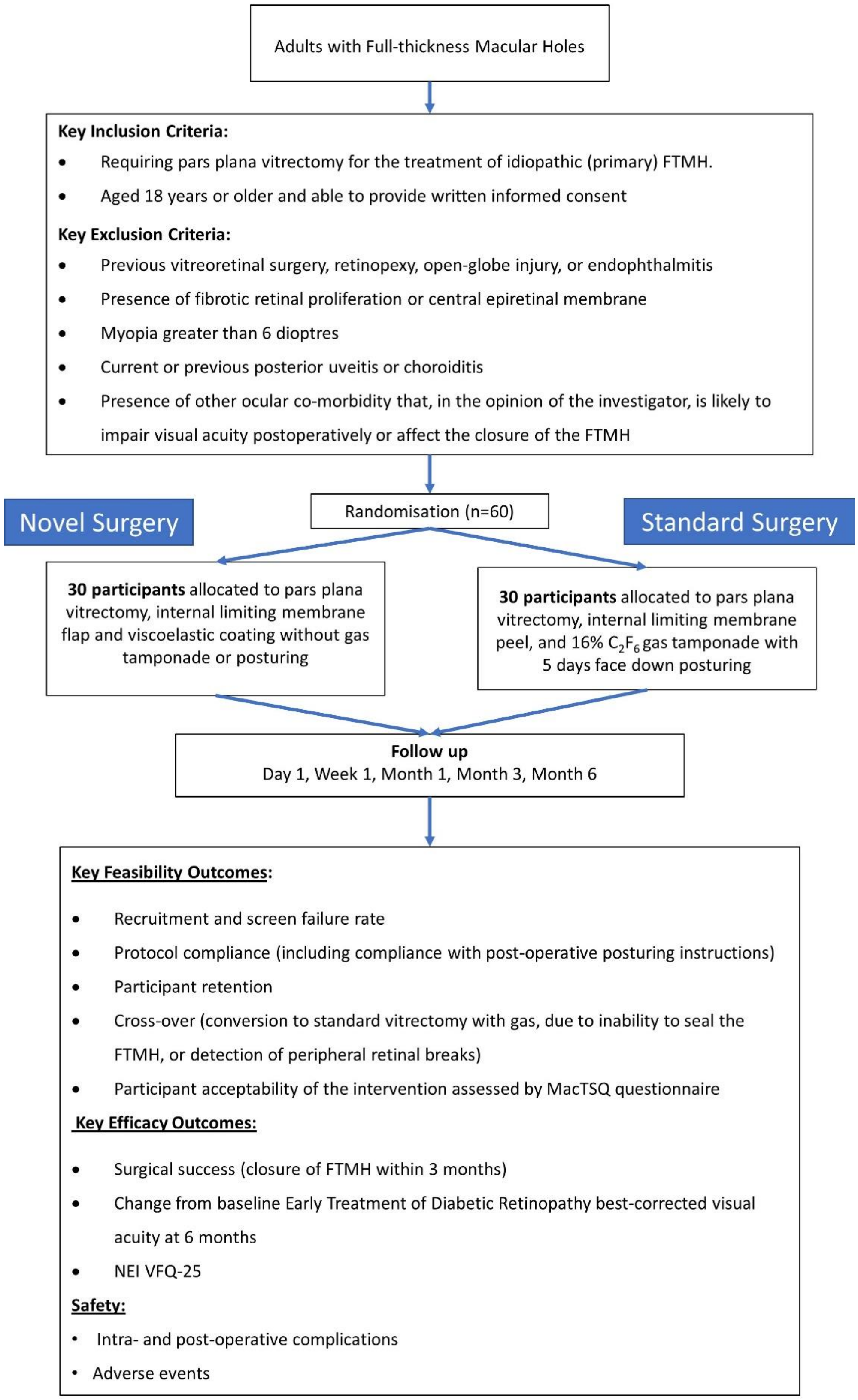

5.4 Study timeline

Feb – Dec ‘23

Study Start

- Local R&D approvals
- Subsite contracting
- PPI – focus group

Nov ‘23 – Oct ‘25

Setup and Recruitment

- Local site training & setup
- Participant recruitment
- PPI - focus group

Oct – Sep ‘26

Follow up

- Patient follow up
- Data cleaning & database lock

May – Oct ‘26

Analysis and dissemination

- Data analysis
- PPI – focus group
- Presentation

5.5 Macular hole surgery

FTMH surgery will be performed by a trial approved consultant vitreoretinal surgeon, most usually the site's Principal Investigator, under local, with or without sedation, or general anaesthesia. Surgery comprises core 23G, 25G, or 27G PPV, induction of posterior vitreous detachment (PVD) if required, and peripheral vitrectomy. Whilst core and peripheral vitrectomy may be performed by a delegated suitably experienced vitreoretinal fellow, all aspects of surgery other than this (including ILM manipulation) must be performed by a trial approved consultant vitreoretinal surgeon.

The surgeon's preferred licensed macular vital stain will be used to stain the ILM.

The gasless surgical technique is based on Stopa *et al*'s [2020] description, wherein a hinged ILM flap is created under heavy liquid, then folded over the FTMH. The surgeon should use decalin if heavy liquid is utilised during surgery, although the use of heavy liquid is optional and based on surgeon experience and preference. A surgical video is provided in Stopa's report:

https://cdn-links.lww.com/permalink/iae/b/iae_2020_04_21_stopa_220-0241_sdc1.mp4.

The only deviation from Stopa's technique is that we aim, where possible, to create a superior flap, so that gravity helps hold it over the macula. Variations may be necessary as sometimes ILM peeling can be difficult to guide precisely (for example, the discarded flap may be used as the free cover if the folded flap does not position as desired).

A layer of Healon Pro® viscoelastic will be applied to the macular area to help hold the flap in place, under the heavy liquid if this has been used. If necessary to retain the flap in position, the flap may be tucked into the hole. Heavy liquid will then be removed. If Healon Pro® is not readily available at a study site, then an equivalent CE-marked OVD licensed for use in the eye may be used, subject to approval from the Sponsor.

The aim is to seal the hole with ILM/viscoelastic, as our mechanistic hypothesis is that fluid flow through the hole maintains patency. However, tamponade will be used if it is not technically possible to occlude the hole using a combination of ILM and OVD.

Those randomised to standard vitrectomy will have the same surgery including a single, approximately 1 disc diameter circular ILM peel centred on the hole, but this will be discarded,

and 16% C_2F_6 tamponade injected into the eye. C_2F_6 has been selected as UK practice surveys indicate it is the most commonly used FTMH tamponade.[12]

Surgery will be video recorded (included in consent). Both arms will be seen 1 day, 1 week, and 1, 3 and 6 months postoperatively.

Repeat surgery with vitrectomy and gas for non-closed holes will be allowed (no further ILM peeling is likely to be possible) and considered a 'failure'.

Other surgical steps will be those routinely used by the operating surgeon, or as needed to deal with any complications, such as cryotherapy and gas for a retinal tear.

For those receiving gas, posture will be face down overnight, then face down by day for 50 minutes/hour, sleeping opposite cheek to pillow, for 5 days. Those not receiving gas do not need to posture.

Cataract surgery is considered in the next section (Cataract Surgery).

5.5.1 Post-operative head positioning (posturing)

Patients in the standard vitrectomy arm will require post-operative posturing. Patients in this arm should be advised to posture face down overnight following surgery, then face down by day for 50 mins/hr, sleeping opposite cheek to pillow, for 5 days. Advise participants to avoid lying on their backs or leaning backwards for 5 days after surgery.

5.5.2 Post-operative management

Prescribe broad-spectrum antibiotic eye drops for at least 1 week after surgery, and topical steroid eye drops for at least 4 weeks postoperatively. Mydriatics are allowed at the surgeon's discretion, for approximately 1-2 weeks. The choice of steroid, antibiotic and mydriatic is at the surgeon's preference, also considering any local policy and the particulars of each participant. Post-operative antiglaucoma medications are also permitted as required, at the surgeon's discretion.

All concomitant medications including post-operative drops should be recorded in the paper source documents and the electronic case report form (eCRF).

Patients should be provided with standard post-operative instructions and those relevant to their case, such as avoiding flying if they have gas in their eye.

5.6 Cataract surgery

Phacoemulsification and IOL implant can be performed prior to vitrectomy in a 'combined procedure' if deemed appropriate, but the decision to undertake phacoemulsification must be made and documented prior to randomisation. If an unplanned cataract occurs during surgery (for example, if there was lens touch and loss of fundal view), then this should be recorded as a deviation. Subsequent cataract surgery is allowed, but deferred until after month 3 unless clinically urgent, in which case it should be recorded as a deviation. Cataract surgery, whether performed as part of a combined procedure or as a standalone procedure, may be undertaken by a suitably qualified and delegated surgeon.

5.7 Data Capture

Information will be collected on paper case report forms (CRF) and transcribed into a corresponding electronic CRF on the King's CTU's 'Macro' Electronic Data Capture (EDC) system. Information from each clinical visit should also be recorded in the participants normal local medical records.

Data will be recorded and securely stored in an electronic data capture system. AEs and serious adverse events (SAEs) will be reported using the Medical Dictionary for Regulatory Activities (MedDRA vers 24.0). Participant narratives will be collated thematically and described.

5.8 OVD risks

In general, OVDs are very well tolerated. Healon PRO® OVD and equivalent OVDs are licenced for intraocular use and systemic side effects are thought to be extremely rare. Allergy to the device is possible, and may present with signs of rash, swelling, and itching. Angio-oedema is

possible but is likewise thought to be extremely rare. Inadvertent injection or migration of OVD into the anterior chamber may cause raised intraocular pressure and should be monitored for at each study visit.

6 STUDY SCHEDULE

Patients diagnosed with a FTMH requiring surgery from one of the study sites will be invited to participate in the trial. Participants must be at least 18 years of age, and require PPV for closure of a FTMH, and not have other vitreoretinal interface abnormalities or a history of prior vitreoretinal surgery. Screening will occur in the ophthalmology clinics and their operation in the normal vitreoretinal theatre at each site. A summary of activities by visit is laid out in Table 1 [Page 8].

Potentially eligible patients will have the standard surgical pathway and trial options described in detail. Written trial participant information sheets will be provided with font size as required. Patients will be given time to read the material, ask questions, and consider the options. This period will typically be at least a few days.

Screening will occur once the participant has given their written informed consent, and surgery will occur at the baseline visit.

Participants will be seen for follow up according to a typical standard of care, with appointments at day 1, week 1, month 1, month 3, and month 6. A summary of the trail procedures at each appointment is included below. Any visits occurring outside of the study schedule should be recorded on a separate 'unscheduled visit' CRF. A return to theatre for any reason felt to be due to the initial study procedure should be recorded and a serious adverse event form completed.

6.1 Screening

Screening activities will not occur until after the participant has provided written informed consent.

Screening includes: demographics; medical and ophthalmic history; NEI VFQ25; full refracted ETDRS BCVA; slit lamp examination; IOP, LOCS2 lens grading; macular optical coherence

tomography (OCT) [6.1.1 Optical Coherence Tomography]; adverse events; concomitant medications.

Phacoemulsification and IOL implant can be performed prior to vitrectomy in a 'combined procedure' if deemed appropriate, but the decision to undertake phacoemulsification should be made and recorded prior to randomisation. Subsequent cataract surgery is allowed but will be deferred until after the month 3 visit (see section 5.6 Cataract Surgery for more details).

Participants will undergo randomisation, by minimisation, prior to surgery but stratified based on planned operation (combined procedure or FTMH surgery alone) and FTMH size based on minimum linear diameter (small <250μm, medium 250-400μm and large >400μm).

6.1.1 Optical Coherence Tomography (OCT)

OCTs will be performed at each study visit except for the day of surgery (it may not be possible to perform the post-operative OCT imaging in the control group until the gas tamponade absorbs). Heidelberg Spectralis spectral domain OCT will be used to capture a macular raster scan, 3x6 mm, consisting of 50 sections with 60 μm spacing, centred on the fovea. An additional radial scan will be captured, consisting of 6 lines centred on the fovea.

The minimum linear diameter and base FTMH diameter will be measured with the calliper tool on the Heidelberg Eye Explorer platform, in the scan with the greatest hole width. Images will be read by the attending clinical investigator to measure the hole minimum linear and basal diameter, and subsequently determine if the hole has closed. Subsequent to local review the anonymised images will be transferred to the Sponsor by NHS email, within 7 days of acquisition, for central masked review. A backup copy of the images will be retained at the study site.

6.1.2 OCT analysis

Images will be uploaded onto the Co-ordinating Centre's Heidelberg Spectralis machine. Images will be read in a masked manner by the Clinical Research Fellow, using the Heidelberg calliper function to measure the minimum linear and base diameter, and hole status (open or closed). Senior masked review will be provided by the CI if required. These masked assessments will be used to describe the trial population (macular hole size) and determine the final anatomic outcome (open or closed at 3 months). Closure of the hole will be determined by the outer

retinal layers being continuous without exposed RPE. Some discontinuity in the inner retina is permissible to still be considered closed.

6.2 Baseline

Macular hole surgery will be performed by a consultant vitreoretinal surgeon and may be performed under local anaesthetic, local anaesthetic with sedation, or general anaesthetic. It will follow the surgical approach detailed in Section 5.5 (macular surgery) and Section 5.6 (cataract surgery).

Phacoemulsification and IOL implant can be performed prior to vitrectomy in a 'combined procedure' if deemed appropriate, but the decision to undertake phacoemulsification will be made prior to randomisation.

Participants will be randomised at the time of screening once the investigator has confirmed eligibility and a decision has been made regarding combined surgery.

Randomisation will be stratified based on planned operation (combined procedure or FTMH surgery alone) and FTMH minimum linear diameter (small <250µm, medium 250-400µm and large >400µm). The measurement of hole size and decision whether or not to operate on the cataract therefore need to occur in advance of randomisation. Also, deciding on cataract surgery prior to randomisation avoids the risk or perception of any bias, in terms of cataract surgery planning.

Participants will be randomised 1:1 through the King's CTU's randomisation platform to either:

- PPV, ILM flap and viscoelastic coating without gas tamponade
- PPV, ILM peel, and 16% C_2F_6 gas tamponade with 5 days face down posturing

For the gasless arm, tamponade should be used if it is not technically possible to occlude the hole using a combination of ILM and OVD.

Surgery will be video recorded (included in consent).

6.3 Day 1, Week 1 & Month 1,3,6 Visits

Both arms will be seen 1 day, 1 week, and 1, 3 and 6 months postoperatively.

Each study visit will include ETDRS VA, IOP, OCT (when gas absorption allows), full ocular examination, LOCS2 lens grading, and recording of adverse events. Compliance with posturing instructions will be assessed by questionnaire at the first two post-operative visits, with a treatment acceptability questionnaire at these and all subsequent visits.

Standard clinic ETDRS VA with appropriate distance and pinhole correction, should be undertaken by unmasked staff at the day 1, week 1, and month 1 visits. Full refracted ETDRS BCVA should be undertaken by an appropriately qualified and delegated masked assessor at the month 3 and 6 visits.

Repeat surgery with vitrectomy and gas for non-closed holes will be allowed (no further ILM peeling is likely to be possible) and considered a 'failure'.

A summary of study visits and activities are shown in Section 1. Compliance with posturing instructions will be assessed by questionnaire at the first two post-operative visits with a bespoke 'service user questionnaire' developed in collaboration with the public contributor panel, postoperatively at day 1 and day 7. Treatment satisfaction will be assessed with the MacTSQ at these and subsequent visits. Feedback from participants and their carers will be collected as free text comments at each visit and included in a qualitative analysis.

6.4 Recruitment timelines and trial duration

The project timeline is included in Study timeline. The trial aims to commence 1st November 2023, allowing 4 months for contracting after intent to fund notification. Set up of all sites is estimated within 6 months.

With the anticipated recruitment rate of 0.5 participants/site/month, this gives an expected final patient recruited date of 23 months after trial start, in October 2025. Follow-up will finish 6 months following the recruitment of the final participant. Data cleaning and lock should take 4 months after final follow up, and data analysis and reporting 6 months after final study visit.

A graph showing projected site set up and recruitment is below.

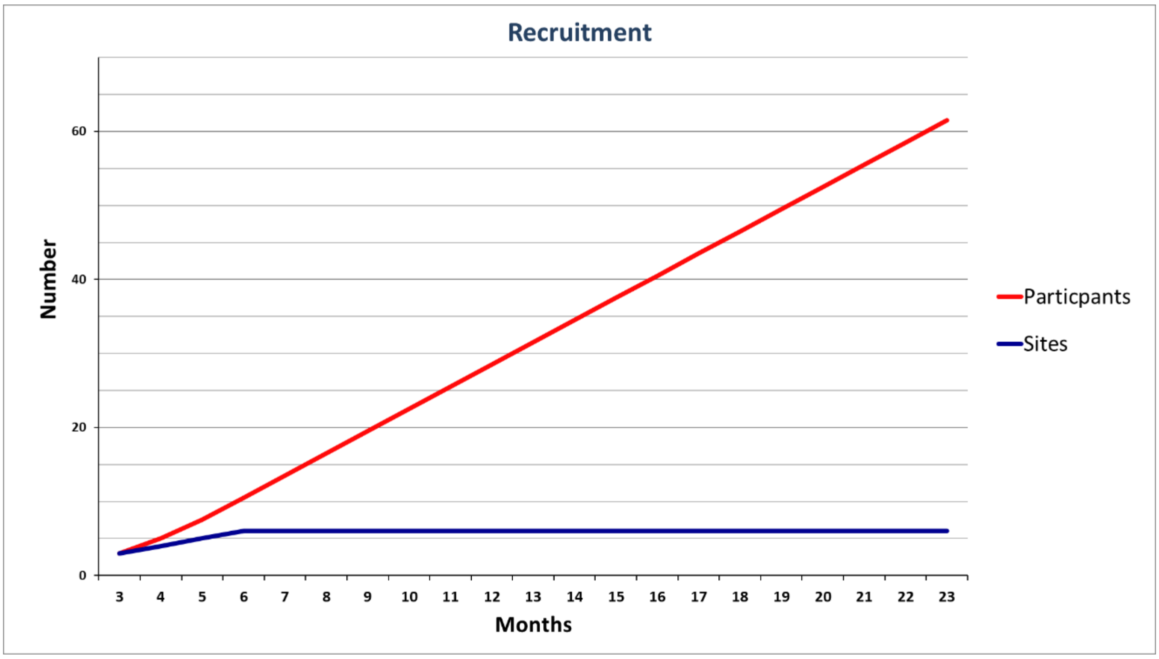

6.5 Study Participation Discontinuation

Participant retention is a key feasibility outcome and will help inform a future study.

Participants have the right to withdraw from the study at any time for any reason. The investigator also has the right to withdraw patients from the study drug in the event of inter-current illness, AE, SAEs, SUSARs, lack of mental capacity, protocol violations, cure, burden of care, administrative reasons, or other reasons.

Participants must not be coerced to remain in the study against their will, but it should be explained that safety monitoring is likely to be more vigilant within the study than outside it. If participants wish to withdraw from the study they should be invited to complete an Exit Visit, which collects the data that is usually acquired at Month 6, but they should not be pressured to accept this invite and can withdraw at any stage without further testing or data collection.

7 CONSENT

A member of the research team will explain the nature of the study to participants, and answer all questions regarding the study, with the aid of a patient information sheet, and give them sufficient time to decide regarding participation. Typically, this is expected to take a few days or

about a week. Participants will be informed that their participation is voluntary, and will be asked to sign an informed consent form prior to any study activities. A copy of the signed informed consent form (ICF) will be provided to all participants.

The study participant information sheet (PIS) will be provided in English, but a hospital interpreting service will be made available for non-English speaking patients. Children and vulnerable populations will not be recruited to this study.

8 ELIGIBILITY CRITERIA

8.1 Inclusion Criteria

- Requiring PPV to treat idiopathic (primary) FTMH.
- 18 years or older
- ETDRS BCVA letter score of 1 or better in the study eye
- Able to provide written informed consent

8.2 Exclusion Criteria

General:

- Hypersensitivity to hyaluronate or other components of Healon Pro® viscoelastic
- Any major illness or major surgical procedure within 4 weeks
- Any other condition that, in the opinion of the investigator, would prevent the participant from granting informed consent or complying with the protocol

Study eye

- Previous vitreoretinal surgery, retinopexy, open-globe injury, or endophthalmitis
- Presence of fibrotic retinal proliferation or central epiretinal membrane (within 1 disc diameter of the fovea)
- Aphakia
- Current or former myopia greater than 6 dioptres
- Current or previous posterior uveitis or choroiditis
- Presence of other ocular co-morbidity that, in the opinion of the investigator, is likely to impair BCVA postoperatively or affect FTMH closure
- Current ocular or periocular infection, other than mild or moderate blepharitis
- Lens or media opacity that preclude adequate retinal assessment and imaging

9 RECRUITMENT & RANDOMISATION

Potentially eligible patients will normally be referred from their optician or other ophthalmologist to hospital vitreoretinal units, for confirmation and treatment of a FTMH. Patients will usually be approached and consented in vitreoretinal clinics.

Potentially eligible patients will have the standard surgical pathway and trial options described in detail. Written trial participant information sheets will be provided with font size as required. Patients will be given time to read the material, ask questions, and consider the options. This period will typically be a few days or about a week.

Participants will not be required to attend more frequent appointments than would otherwise occur during standard of care. A contact number for the local research team will be included in all participant information sheets, should the participants need to contact the team with issues or concerns arising during the study.

A screening log of patients approached for the study will be kept on a secure computer in the research office.

Participants will be randomised at the time of screening once the investigator has confirmed eligibility and a decision has been made regarding combined surgery.

Participants will undergo randomisation by minimisation to assign treatment prior to surgery. They will be stratified based on planned operation (combined procedure or FTMH surgery alone) and FTMH size (small, medium and large). Phacoemulsification and IOL implant can be performed prior to vitrectomy in a 'combined procedure' if deemed appropriate, but the decision to undertake phacoemulsification should be made prior to randomisation.

A web based bespoke randomisation system will be created in collaboration with the trial statisticians and the CI and maintained by the King's Clinical Trials Unit (KCTU) for the duration of the project. It will be hosted on a dedicated server within KCL. The KCTU will provide the Trial Managers with a Data Management Plan for the web based KCTU Randomisation System, once the system is made live and ready for use. That document will be filed in the Trial Master File.
The CI or delegate will request usernames and passwords from the KCTU. System access will be strictly restricted through user-specific passwords to the authorised research team members. It is a legal requirement that passwords to the randomisation system are not shared, and that only those authorised to access the system are allowed to do so. If new staff members join the study,

a user-specific username and password must be requested via the CI or delegate (e.g Trial Manager) from the KCTU team and a request for access to be revoked must be requested when staff members leave the project. Study site staff experiencing issues with system access or functionality should contact the CI or delegate.

10 STATISTICAL METHODS

10.1 Sample size

For feasibility and pilot studies, sample sizes between 24 and 50 have been recommended to estimate a chosen parameter.[21,22] Using a 1:1 treatment to control ratio, a total sample size of 60 would be sufficient to estimate the standard deviation of the outcome of at least 28 treated participants per group, with 7% attrition. We aim to estimate the screening success (assumed as 70%; 95% confidence interval [95% CI]:60-80) from approximately 105 potentially eligible participants.

Of the 60 participants, we will enrol at least 16 with small FTMHs (<250 μm minimum linear diameter), 16 medium (250-400 μm), and 16 large (>400 μm).

We aim for approximately six sites, starting with King's College Hospital and Sunderland Eye Infirmary, where the clinical co-applicants work, to 'iron-out' any protocol or practical issues. Together, these two sites perform approximately 244 FTMH operations annually, with approximately 12 eligible patients per month. The anticipated subsequent sites are Bristol Eye Hospital, Hull and East Yorkshire Eye Hospital, Sussex Eye Hospital, and St Paul's Eye Unit Liverpool. Assuming 0.5 recruits per month per site, we project recruitment taking 23 months.

10.2 Statistical Analysis

A statistical analysis plan will be written by the trial statistician and approved prior to the first participant being recruited. Description of baseline characteristics will be provided for the two arms using mean and standard deviation for approximately normally distributed continuous variables, or median and interquartile range for non-normal continuous variables (skewed). Categorical variables will be reported as count and percentage with the 95% CI. Baseline variables will include age, sex, BCVA, pre-operative lens status, laterality, minimum linear and basal hole diameter, presence of PVD, refraction, and duration of symptoms.

The proportion of patients who successfully screen will be reported with 95% CI computed by the exact binomial method, as will all outcomes assessing feasibility except for recruitment rate, which will be reported per site and per month. In addition to summary statistics of the secondary outcomes (similarly to the primary and baseline outcomes), all harms and withdrawals will be reported with 95% CIs.

An exploratory efficacy analysis will be intent-to-treat and include all randomised participants. A secondary analysis will compare those who had gasless versus standard surgery, irrespective of allocation, to investigate the effect of cross-over. The safety population will include all participants who underwent randomisation.

The proportion of participants with surgical success and mean BCVA (change from baseline, final and area under the curve [AUC]) will be compared between groups at 6 months (Fisher's exact test and Mann-Whitney respectively), with subgroup analyses based on lens status and FTMH size. A subgroup analysis will be performed, with the trial population split into each surgeon's first five cases, vs all those subsequently. This will inform a descriptive analysis of the learning curve for the surgical technique, with an intention to perform a cumulative sum curve if an individual surgeon performs sufficient cases to inform the analysis. For the AUC analysis BCVA will often be below 1 ETDRS letter when gas is in the eye, so ETDRS acuity will be converted to logarithm of the minimum angle of resolution (logMAR) with assignment of logMAR values to count fingers or worse[23]:

Count Fingers at 2ft: logMAR +1.85

Hand Motions at 2ft: logMAR +2.3

Visual acuity of 'Light Perception' or 'No Light Perception' will not be converted and will be reported separately.

Adherence to posturing and treatment acceptability will be described.

The MacTSQ has been validated for use in macular diseases requiring drug administration and consists of two subscales: information provision/convenience and treatment satisfaction.[24] The individual scores from each subscale as well as the composite score will be analysed based on change from the first time point (paired sample t-test) and between groups (analysis of variance model).

Quantifiable findings from the MacTSQ will be analysed using descriptive statistics. Open ended responses will be analysed for descriptive and analytic accounts using thematic analysis and a constant comparison approach. Analysis will pay particular attention to key factors that impact

on patient experiences both positively and negatively, and will integrate quantifiable data from the MacTSQ in the analysis to investigate patterns of experience within the participant population. Qualitative findings will also be discussed in detail with the public contributor panel to consider how the patient experience can be optimised to support retention and overall satisfaction.

Participant narratives will be collated thematically and described.

11 PATIENT AND PUBLIC INVOLVEMENT (PPI)

The public (patient and carer) contributor panel will consist of at least six people who have previously undergone macular hole surgery and two of their partners or relatives who cared for them during the post-operative period.

A PPI lead will co-ordinate PPI activities throughout the study. Our PPI budget has been calculated through the INVOLVE calculator, and funds her to set the objectives and format of our planned PPI meetings, prepare the topic guide, give a post meeting debrief, and pay our public contributor panel volunteers for their travel and time. Remuneration for the panel volunteers will be discussed individually and options given to ensure that the preferred method is used, given each participant's tax status. Consideration will be made for holding sessions online if preferred.

The public contributor panel will review the PIS, assist with protocol design, collaborate in the design of bespoke service user questionnaire, serve as a 'sounding board' for PPI issues, advise on how to boost recruitment and retention, comment on how best to communicate and disseminate the results to the study participants and wider community, and help us to determine thresholds of success to be used in a definitive trial. The outcomes from these sessions will be collated and shared with the study team.

A quarterly newsletter will be sent to the public contributor panel from the study team to keep them informed of the progress of the trial.

The public contributor panel will be central to determining an acceptable non-inferiority margin for a pivotal trial, which in turn determines whether a pivotal trial should go head.

12 FUNDING AND SUPPLY OF EQUIPMENT

The study funding has been reviewed by the KCH R&D Office, and deemed sufficient to cover the requirements of the study.

The research costs for the study have been supported a National Institute for Health and Care Research (NIHR) Research for Patient Benefit Grant (GRANT NO: NIHR204994).

No additional equipment is required for the surgery; Healon PRO® or equivalent OVD is part of regular stock in an NHS ophthalmic theatre. Other required equipment for ophthalmic assessments is also as standard for eye clinics.

13 DATA HANDLING AND MANAGEMENT

The Chief Investigator (CI) will act as custodian for the trial data. Patient data will be pseudonymised. All pseudonymised data will be stored on a password protected computer. All trial data will be stored in line with the Medicines for Human Use (Clinical Trials) Amended Regulations 2006 and the Data Protection Act and archived in line with the Medicines for Human Use (Clinical Trials) Amended Regulations 2006, as defined in the King's Health Partners Clinical Trials Office Archiving Standard Operating Procedures (SOPs).

Paper source data worksheets will be used alongside normal clinical visit data recorded on the local electronic medical record (EMR). There will be a corresponding, online, secure, eCRF. Sites will transfer data from the trial source data worksheets to the eCRF. Paper source worksheets will be stored in a secure, locked, office at each site. The data will reside on an online, secure, trial database; King's CTU 'Macro' EDC.

- The Sponsor will archive trial data once data analysis is complete via a secure database (http://www.ironmountain.co.uk), for 10 years.
- The CI will act as custodian for the trial data.

13.1 Macro EDC

A web based electronic data capture (EDC) system will be designed, using the InferMed Macro 4 system.

Data entry

The EDC will be created in collaboration with the trial analysts and the CI and maintained by the King's Clinical Trials Unit for the duration of the project. It will be hosted on a dedicated server within KCL.

Source data will be entered by site staff, typically within 7 days of data collection by authorised staff onto the EDC by going to www.ctu.co.uk and clicking the link to access the MACRO 4 EDC system. A full audit trail of data entry and any subsequent changes to entered data will be automatically date and time stamped, alongside information about the user making the entry/changes within the system.

Security

The CI or delegate will request usernames and passwords from the KCTU. Database access will be strictly restricted through user-specific passwords to the authorised research team members. It is a legal requirement that passwords to the EDC are not shared, and that only those authorised to access the system are allowed to do so. If new staff members join the study, a user-specific username and password must be requested via the CI or delegate (e.g Trial Manager) from the KCTU team and a request for access to be revoked must be requested when staff members leave the project. Study site staff experiencing issues with system access or functionality should contact the CI or delegate (e.g., Trial Manager) in the first instance.

Participant initials and possibly date of birth will be entered on the EDC. Whereas NHS number, email addresses, participant names, addresses, and full postcodes will not be entered into the EDC system. No data will be entered onto the EDC system unless a participant has signed a consent form to participate in the trial.

Data Quality Processes

The CI team will undertake appropriate reviews of the entered data, in consultation with the project analyst, for the purpose of data cleaning and will request amendments as required. No data will be amended independently of the study site responsible for entering the data.

The KCTU will provide the study team with Data management plan for Elsevier InferMed MACRO EDC once the system is made live and ready for use.

Database Lock

At the end of the trial, the site PI will review all the data for each participant and provide electronic sign-off, to verify that all the data are complete and correct. At this point, all data can be formally locked for analysis.

Upon request, KCTU will provide a copy of the final exported dataset to the CI in .csv format and the CI will onward distribute as appropriate.

13.2 KCTU Randomisation

A web based randomisation system will be designed, using the bespoke KCTU randomisation system. The randomisation system will be created in collaboration with the trial analysts and the CI and maintained by the King's Clinical Trials Unit for the duration of the project. It will be hosted on a dedicated server within KCL.

Randomisation will be at the level of the individual using the method of minimisation prior to surgery., They will be stratified based on minimisation factors of planned operation (combined procedure or FTMH surgery alone) and FTMH size (small, medium and large).

Data entry

Randomisation will be undertaken by site staff, by authorised staff onto the randomisation system by going to www.ctu.co.uk and clicking the link to access the randomisation system. A full audit trail of data entry will be automatically date and time stamped, alongside information about the user making the entry within the system.

Security

The CI or delegate will request usernames and passwords from the KCTU. System access will be strictly restricted through user-specific passwords to the authorised research team members. It is a legal requirement that passwords to the randomisation system are not shared, and that only those authorised to access the system are allowed to do so. If new staff members join the study, a user-specific username and password must be requested via the CI or delegate (e.g., Trial Manager) from the KCTU team and a request for access to be revoked must be requested when staff members leave the project. Study site staff experiencing issues with system access or functionality should contact the CI or delegate (e.g., Trial Manager) in the first instance.

Participant initials and date of birth will be entered on the randomisation system. Whereas NHS number, email addresses, participant names, addresses, and full postcodes will not be entered into the randomisation system. No data will be entered onto the randomisation system unless a participant has signed a consent form to participate in the trial.

Data Quality Processes

The CI team will undertake appropriate reviews of the entered data, in consultation with the project analyst for the purpose of data cleaning. No data can be amended in the system, however CI or delegate (e.g., Trial Manager) may request King's Clinical Trials Unit to add notes against individual subject entries to clarify data entry errors.

Database Lock

Upon request, KCTU will provide a copy of the final exported dataset to the CI in .csv format and the CI will onward distribute as appropriate.

13.3 Trial Management

King's College Hospital will sponsor the study and be the co-ordinating site, supported by the King's CTU, which provides randomisation, electronic data capture, data management and statistical analysis. The CTU is a UKCRC-accredited academic unit supporting clinical trial design, conduct and dissemination (http://www.ctu.co.uk/). It has one of the largest trial portfolios in the UK and has experience of supporting over 250 clinical trials, including 14 ophthalmology trials.

The Trial Management Group will comprise all co-applicants and will meet monthly. Day-to-day running of the trial will be undertaken by the Clinical Research Fellow, and CI, who will meet at least weekly.

A Trial Steering Committee (TSC) will be convened in accordance with National Institute for Health and Care Research (NIHR) Guidelines, and a TSC Terms of Reference, to review the protocol prior to trial commencement and then meet at least yearly. Similarly, a Data Monitoring and Ethics Committee (DMEC) will be convened per NIHR guidance and tasked to review safety data at intervals they determine as appropriate, consistent with NIHR guidance and a trial specific DMEC Charter.

A physical trial master file (TMF) will be maintained at each site and contain all correspondence with and reports/letters from investigators, research ethics committees (REC), and other regulatory authorities, an up-to-date version of the study protocol, and original source data worksheets.

13.4 Ethics / Regulatory Approvals

All study partners and sites will ensure that all activities comply with all national legal and ethical requirements.

The team are familiar with conducting trials compliant with trial and data protection regulations and have extensive and robust SOPs to ensure compliance. They have worked together on this application and have agreed responsibilities. There is no research on animals or non-human primates and no collection of human tissue.

All approvals including national permissions will be in place at participating centres prior to participant enrolment. All trial investigators are required to undertake Good Clinical Practice (GCP) training, maintain site files, and these will be in order before the Sponsor agrees to open the study. Strict pharmacovigilance procedures will be in place with respect to AE and SAE reporting. The level of risk will be assessed in accordance with the Sponsor's standard processes.

The study will be submitted to and receive a favourable opinion for a UK Research Ethics Committee (REC) and will require authorisation for each participating site prior to enrolling participants. The CI will prepare a final report in accordance with NIHR requirements.

As the OVD has CE marking as a medical device and has authorisation to "be used to efficiently separate and control ocular tissues", we believe the trial's use of OVD falls within this description and anticipate that the trial is unlikely to be a CTIMP. Heavy liquid is likewise used within its indication for use.

The study will be registered on ClinicalTrials.gov prior to commencement and the protocol will be made publicly available before data lock.

14 PEER AND REGULATORY REVIEW

The trial has been peer reviewed by the National Institute for Health and Care Research (NIHR) through a successful funding bid. The study has been subject to independent external peer reviewed in accordance with the requirements outlined by King's College Hospital R&I. The study has been deemed to be an observational study design, by the KCH R&D department. The OVD is being used within its CE mark and the outcome of interest is the new surgical technique. The study will be reviewed by a UK Research Ethics Committee.

15 ADVERSE EVENTS AND INCIDENT REPORTING

15.1 Definitions of Adverse Events

Term	Definition
Adverse Event (AE)	Any untoward medical occurrence in a patient or study participant, which does not necessarily have a causal relationship with the intervention/treatment/procedure involved.
Serious Adverse Event (SAE).	Any adverse event that:<br>• results in death,<br>• is life- threatening*,<br>• requires hospitalisation or prolongation of existing hospitalisation**,<br>• results in persistent or significant disability or incapacity, or<br>• consists of a congenital anomaly or birth defect
*A life- threatening event, this refers to an event in which the participant was at risk of death at the time of the event; it does not refer to an event which hypothetically might have caused death if it were more severe.<br>** Hospitalisation is defined as an in-patient admission, regardless of length of stay. Hospitalisation for pre-existing conditions, including elective procedures do not constitute an SAE.	

15.2 Assessments of Adverse Events

Each adverse event will be assessed for severity, causality, seriousness and expectedness as described below.

15.2.1 Severity

Category	Definition
Mild	The adverse event does not interfere with the participant's daily routine, and does not require further procedure; it causes slight discomfort
Moderate	The adverse event interferes with some aspects of the participant's routine, or requires further procedure, but is not damaging to health; it causes moderate discomfort
Severe	The adverse event results in alteration, discomfort or disability which is clearly damaging to health

15.2.2 Causality

The assessment of relationship of adverse events to the procedure is a clinical decision based on all available information at the time of the completion of the case report form. Causality will be determined by the attending site investigator. The Sponsor can query causality with the site investigator, but the local PI will make the final determination. The Sponsor can 'upgrade' relatedness, for example, from possibly to probably.

The following categories will be used to define the causality of the adverse event:

**Category**	**Definition**
Definitely:	There is clear evidence to suggest a causal relationship, and other possible contributing factors can be ruled out.
Probably:	There is evidence to suggest a causal relationship, and the influence of other factors is unlikely
Possibly	There is some evidence to suggest a causal relationship (e.g. the event occurred within a reasonable time after administration of the study procedure). However, the influence of other factors may have contributed to the event (e.g. the participant's clinical condition, other concomitant events).
Unlikely	There is little evidence to suggest there is a causal relationship (e.g. the event did not occur within a reasonable time after administration of the study procedure). There is another reasonable explanation for the event (e.g. the participant's clinical condition).
Not related	There is no evidence of any causal relationship.

Causality will be determined in relation to both the device (the OVD viscoelastic used to secure the ILM flap), and the surgery (any of the steps undertaken during macular hole surgery, and cataract surgery if that also applies)

15.2.3 Expectedness

Category	Definition
Expected	An adverse event which is consistent with the available information about the intervention/treatment/procedure in use in this study.
Unexpected	An adverse event which is not consistent with the available information about the intervention/treatment/procedure in use in this study*

* this includes listed events that are more frequently reported or more severe than previously reported. The list of expected AEs is defined by the OVD manufacturer's product brochure.

15.3 Procedures for recording adverse events

All Adverse events will be recorded in the CRF following consent until the patient completes the study.

15.4 Procedures for recording and reporting serious adverse events

All serious adverse events (SAEs) will be recorded in the medical records and the CRF.

All SAEs (except those specified in section 15.5 as not requiring reporting to the Sponsor) must be recorded on a serious adverse event (SAE) form. The PI or designated individual will complete an SAE form and it should be emailed to both the KCH R&D office (kch-

tr.research@nhs.net) and to the Chief Investigator within 1 working day of becoming aware of the event.

Where the event is unexpected and thought to be related to the procedure this must be reported by the Investigator to the REC and Health Research Authority, using the SAE Report form for non-CTIMPs (available from the HRA website) within 15 days.

All reporting to King's College Hospital NHS Foundation Trust should be by e-mail giving as much information about the incident as possible, and should be signed by the PI or Co-investigator.

The Sponsor will undertake an initial review of the information. Events will be followed up until resolution, and any appropriate further information will be sent by the research team in a timely manner.

15.5 Serious Adverse Events that do not require reporting

Failure of anatomical closure of the macular hole should be recorded on the CRF and not as an AE/SAE, unless it requires a return to theatre in which instance it should reported as an SAE.

Intraocular pressure elevation to less than 25.0 mmHg on the day 1 or week 1-2 post-operative visit does not need to be recorded as an AE or SAE, as this is common after vitrectomy, but the use of any pressuring lowering medicines should be recorded on the CRF. Cataract requiring surgery should be recorded as an AE.

Peripheral retinal breaks that occur during surgery should be recorded as an AE,. Retinal breaks detected subsequently should be recorded as an AE.

15.6 Reporting Urgent Safety Measures

If any urgent safety measures are taken the CI/ PI shall immediately and in any event no later than 3 days from the date the measures are taken, give written notice to the relevant REC, Health Research Authority and R&I office of the measures taken and the circumstances giving rise to those measures.

15.7 Protocol deviations and notification of protocol violations

A deviation is usually an unintended departure from the expected conduct of the study protocol/SOPs, which does not need to be reported to the Sponsor. The CI will monitor protocol deviations.

A protocol violation is a breach which is likely to effect to a significant degree –

(a) the safety or physical or mental integrity of the participants of the study; or

(b) the scientific value of the study.

The CI and R&D Office should be notified immediately of any case where the above definition applies during the study conduct phase.

Flow Chart for SAE reporting

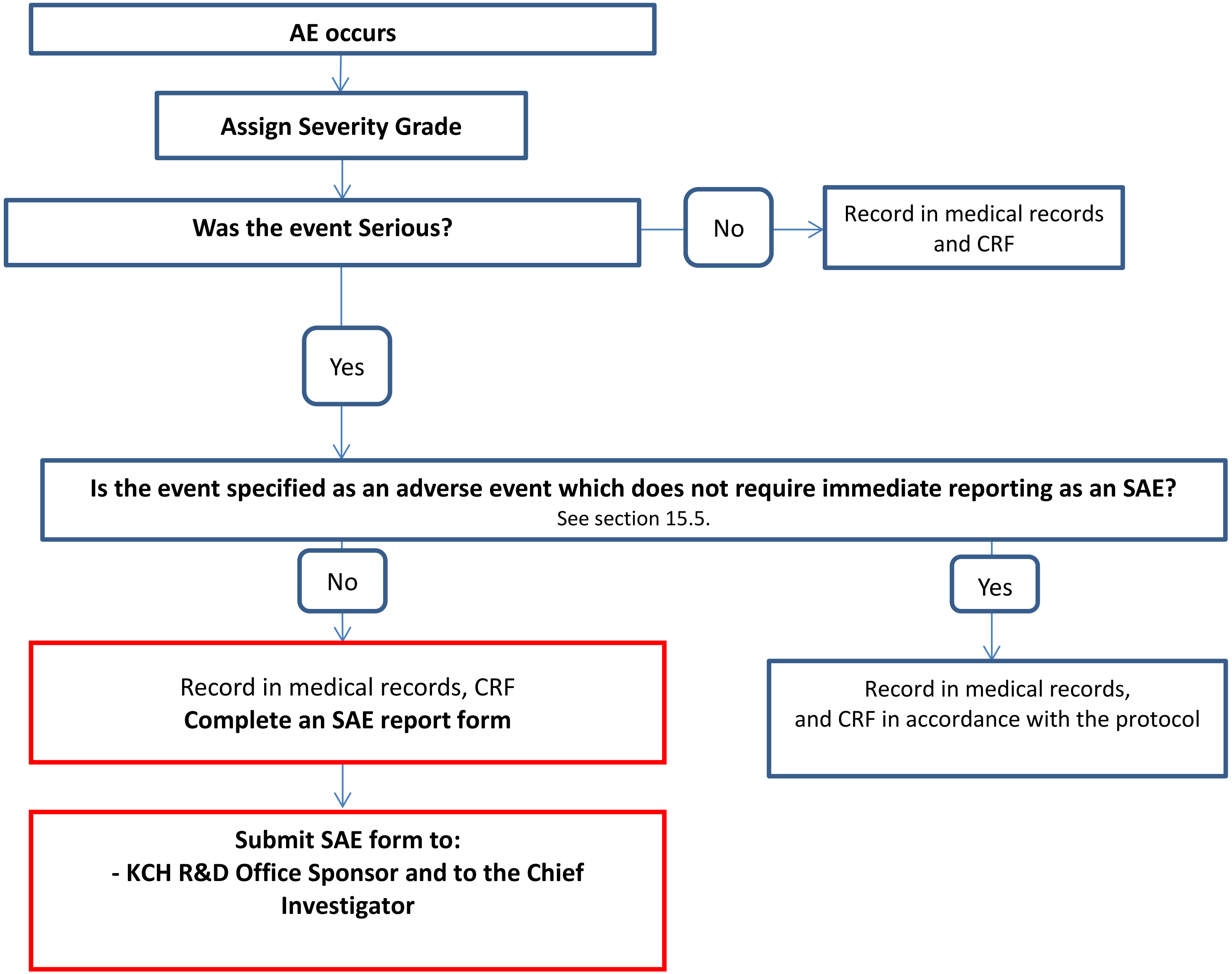

15.8 Trust incidents and near misses

An incident or near miss is any unintended or unexpected event that could have or did lead to harm, loss or damage that contains one or more of the following components:

a. It is an accident or other incident which results in injury or ill health.
b. It is contrary to specified or expected standard of patient care or service.
c. It places patients, staff members, visitors, contractors or members of the public at unnecessary risk.
d. It puts the Trust in an adverse position with potential loss of reputation.
e. It puts Trust property or assets in an adverse position or at risk.

Incidents and near misses must be reported to the Trust through DATIX or other similar system, as soon as the individual becomes aware of them.
A reportable incident is any unintended or unexpected event that could have or did lead to harm, loss or damage that contains one or more of the following components:

a) It is an accident or other incident which results in injury or ill health.
b) It is contrary to specified or expected standard of patient care or service.
c) It places patients, staff members, visitors, contractors or members of the public at unnecessary risk.
d) It puts the Trust in an adverse position with potential loss of reputation.
e) It puts Trust property or assets in an adverse position or at risk of loss or damage.

16 MONITORING AND AUDITING

The CI will ensure there are adequate quality and number of monitoring activities conducted by the study team. This will include adherence to the protocol, procedures for consenting and ensure adequate data quality.

The CI will inform the sponsor should he/she have concerns which have arisen from monitoring activities, and/or if there are problems with oversight/monitoring procedures.

17 TRAINING

The CI will review and provide assurances of the training and experience of all staff working on this study. Appropriate training records will be maintained in the study files.
Study surgeons, performing the macular hole surgery, will be NHS consultant vitreoretinal surgeons at least 3 years post-certificate of completion of training. They will be routinely performing macular hole surgery with ILM flaps. Prospective surgeons will be required to provide a video of their flap technique, as part of the site selection process.

There will be a surgical training video and written protocol, that will both be provided in advance of each site's initiation visit. Each new surgeon's first five on-trial surgical videos will be reviewed with an assigned senior mentor. Technical success will be classified as complete (flap created as planned), partial (flap inserted into the hole), and failure (flap not secured in situ and therefore gas tamponade required).

Only the refracted best corrected visual acuity and OCT assessors are masked (there is no sham for gas), and the technical and anatomic success rates and study eye adverse events (AEs) will be regularly monitored at a site level by the CI. No fixed criteria will be set to prompt surgeon re-evaluation, as there will be material variations in case mix, and chance may impact on closure rates. Nonetheless, if a surgeon appears as an outlier, the CI, mentor, and surgeon will convene a meeting to consider how best to support the surgeon, whilst maximising participant safety.

The site's lead VA examiner can delegate clinic ETDRS VA testing to suitably trained clinic staff who, unlike those testing the Full Refracted ETDRS BCVA, but these staff members must appear on the delegation log and require at least GCP 'light' training.

18 INTELLECTUAL PROPERTY

There is not anticipated to be any intellectual property rights arising from this project.

19 INDEMNITY ARRANGEMENTS

KCH will provide NHS indemnity cover for negligent harm, as appropriate and is not in the position to indemnify for non-negligent harm. NHS indemnity arrangements do not extend to non-negligent harm and NHS bodies cannot purchase commercial insurance for this purpose; it cannot give advance undertaking to pay compensation when there is no negligence attributable to their vicarious liability. The Trust will only extend NHS indemnity cover for negligent harm to

its employees, both substantive and honorary, conducting research studies that have been approved by the R&D Department. The Trust cannot accept liability for any activity that has not been properly registered, and Trust approved. Potential claims should be reported immediately to the R&I Office

20 ARCHIVING

The sponsor will archive the trial data, both paper CRFs and the electronic database, via Iron Mountain for 10 years.

21 PUBLICATION AND DISSEMINATION POLICY

It is intended that the protocol for this study is published in a peer reviewed journal, or deposited on a public registry, to reduce the risk or perception of publication bias.

Working with the study team the public (patient and carer) contributor panel will lead on dissemination of the trial's outputs, including participants' reported outcomes, to inform a wider audience. This will include producing a lay summary report for patients, shared through eye charities and hospital news channels as well as information hosted on appropriate websites and social media. Participants will be informed of the trial's progress via a regular newsletter.

All participants will be invited to receive a copy of the trial results (unless they wish not to).

Vitreoretinal surgeons are key stakeholders, as they will be the ones to recruit onto any subsequent pivotal trial, and drive adoption if gasless macular hole surgery is proven efficacious. Accordingly, the study results will be presented at national and international retinal conferences, and submitted for publication in a peer-reviewed clinical journal. Authorship will be determined by the CI, in discussion with the Co-Investigator, to reflect the relative contribution of staff to the design, execution, analysis and write-up of the results. It is anticipated that the Co-Investigators will be a named authors. The trial design, methodology and results may contribute to the doctoral thesis of students supervised by the CI.

22 REFERENCES

1. Johnson RN, Gass JDM. Idiopathic Macular Holes. *Ophthalmology*. 1988;95(7):917-924. doi:10.1016/s0161-6420(88)33075-7
2. McCannel CA, Ensminger JL, Diehl NN, Hodge DN. Population-based incidence of macular holes. *Ophthalmology*. Jul 2009;116(7):1366-9. doi:10.1016/j.ophtha.2009.01.052
3. Mitchell P, Smith W, Chey T, Wang JJ, Chang A. Prevalence and Associations of Epiretinal Membranes. *Ophthalmology*. 1997;104(6):1033-1040. doi:10.1016/s0161-6420(97)30190-0
4. Rahmani B, Tielsch JM, Katz J, et al. The Cause-specific Prevalence of Visual Impairment in an Urban Population. *Ophthalmology*. 1996;103(11):1721-1726. doi:10.1016/s0161-6420(96)30435-1
5. Wang S, Xu L, Jonas JB. Prevalence of full-thickness macular holes in urban and rural adult Chinese: the Beijing Eye Study. *Am J Ophthalmol*. Mar 2006;141(3):589-91. doi:10.1016/j.ajo.2005.10.021
6. Sen P, Bhargava A, Vijaya L, George R. Prevalence of idiopathic macular hole in adult rural and urban south Indian population. *Clin Exp Ophthalmol*. Apr 2008;36(3):257-60. doi:10.1111/j.1442-9071.2008.01715.x
7. Parravano M, Giansanti F, Eandi CM, Yap YC, Rizzo S, Virgili G. Vitrectomy for idiopathic macular hole. *Cochrane Database Syst Rev*. 2015;(5)doi:10.1002/14651858.CD009080.pub2
8. Jackson TL, Donachie PHJ, Sparrow JM, Johnston RL. United Kingdom National Ophthalmology Database study of vitreoretinal surgery: report 2, macular hole. *Ophthalmology*. Mar 2013;120(3):629-634. doi:10.1016/j.ophtha.2012.09.003
9. Yamashita T, Sakamoto T, Yamashita T, et al. Individualized, spectral domain-optical coherence tomography-guided facedown posturing after macular hole surgery: minimizing treatment burden and maximizing outcome. *Retina*. Jul 2014;34(7):1367-75. doi:10.1097/iae.0000000000000087
10. Hu Z, Xie P, Ding Y, Zheng X, Yuan D, Liu Q. Face-down or no face-down posturing following macular hole surgery: a meta-analysis. *Acta Ophthalmol*. Jun 2016;94(4):326-33. doi:10.1111/aos.12844
11. Tsai HR, Chen TL, Chang CY, Huang HK, Lee YC. Face-Down Posture versus Non-Face-Down Posture following Large Idiopathic Macular Hole Surgery: A Systemic Review and Meta-Analysis. *J Clin Med*. Oct 24 2021;10(21)doi:10.3390/jcm10214895
12. Steel DH, Donachie PHJ, Aylward GW, Laidlaw DA, Williamson TH, Yorston D. Factors affecting anatomical and visual outcome after macular hole surgery: findings from a large prospective UK cohort. *Eye (Lond)*. Jan 2021;35(1):316-325. doi:10.1038/s41433-020-0844-x
13. Kontos A, Tee J, Stuart A, Shalchi Z, Williamson TH. Duration of intraocular gases following vitreoretinal surgery. *Graefes Arch Clin Exp Ophthalmol*. Feb 2017;255(2):231-236. doi:10.1007/s00417-016-3438-3
14. Kanclerz P, Grzybowski A. Complications Associated with the Use of Expandable Gases in Vitrectomy. *J Ophthalmol*. 2018;2018:8606494. doi:10.1155/2018/8606494
15. Cekic O, Ohji M. Intraocular gas tamponades. *Semin Ophthalmol*. Mar 2000;15(1):3-14. doi:10.3109/08820530009037846
16. Wang CP, Huang EJ, Kuo CN, Lai CH. Deep vein thrombosis due to continuous prone positioning after retinal detachment surgery. *Taiwan J Ophthalmol*. Apr-Jun 2016;6(2):96-97. doi:10.1016/j.tjo.2015.05.002
17. Morimoto E, Shimada Y, Sugimoto M, Mizuguchi T, Tanikawa A, Horiguchi M. Adherence to face-down and non-supine positioning after macular hole surgery. *BMC Ophthalmol*. 2018/12/14 2018;18(1):322. doi:10.1186/s12886-018-0979-8
18. Stopa M, Ciesielski M, Rakowicz P. Macular Hole Closure Without Endotamponade Application. *Retina (Philadelphia, Pa)*. 2020;

19. Chakrabarti M, Benjamin P, Chakrabarti K, Chakrabarti A. Closing macular holes with “macular plug” without gas tamponade and postoperative posturing. *Retina*. 2017;37(3):451-459.
20. Zhu D, Ma B, Zhang J, et al. Autologous blood clot covering instead of gas tamponade for macular holes. *Retina*. 2020;40(9):1751-1756.
21. Sim J, Lewis M. The size of a pilot study for a clinical trial should be calculated in relation to considerations of precision and efficiency. *J Clin Epidemiol*. 2012;65(3):301-308.
22. Julious SA. Issues with number needed to treat. *Stat Med*. 2005;24(20):3233-3235.
23. Schulze-Bonsel K, Feltgen N, Burau H, Hansen L, Bach M. Visual acuities "hand motion" and "counting fingers" can be quantified with the freiburg visual acuity test. *Invest Ophthalmol Vis Sci*. Mar 2006;47(3):1236-40. doi:10.1167/iovs.05-0981
24. Mitchell J, Bradley C. Design and development of the MacTSQ measure of satisfaction with treatment for macular conditions used within the IVAN trial. *Journal of Patient-Reported Outcomes*. 2018;2(1):1-9.

Signed: (Tim Jackson, Chief Investigator)

Date:

23 APPENDICES
